# Efficient derivation of functional astrocytes from human induced pluripotent stem cells (hiPSCs)

**DOI:** 10.1371/journal.pone.0313514

**Published:** 2024-12-04

**Authors:** Balazs Szeky, Veronika Jurakova, Eliska Fouskova, Anita Feher, Melinda Zana, Vivien Reka Karl, Janos Farkas, Maria Bodi-Jakus, Martina Zapletalova, Shashank Pandey, Radek Kucera, Jan Lochman, Andras Dinnyes

**Affiliations:** 1 BioTalentum Ltd, Godollo, Hungary; 2 Department of Biochemistry, Faculty of Science, Masaryk University, Brno, Czech Republic; 3 Department of Pharmacology and Toxicology, Faculty of Medicine in Pilsen, Charles University, Pilsen, Czech Republic; 4 Department of Immunochemistry Diagnostics, University Hospital Pilsen, Pilsen, Czech Republic; 5 Laboratory of Neurobiology and Pathological Physiology, Institute of Animal Physiology and Genetics, Czech Academy of Sciences, Brno, Czech Republic; 6 Department of Physiology and Animal Health, Institute of Physiology and Animal Nutrition, Hungarian University of Agriculture and Life Sciences, Godollo, Hungary; University Hospital Zurich: UniversitatsSpital Zurich, SWITZERLAND

## Abstract

Astrocytes are specialized glial cell types of the central nervous system (CNS) with remarkably high abundance, morphological and functional diversity. Astrocytes maintain neural metabolic support, synapse regulation, blood-brain barrier integrity and immunological homeostasis through intricate interactions with other cells, including neurons, microglia, pericytes and lymphocytes. Due to their extensive intercellular crosstalks, astrocytes are also implicated in the pathogenesis of CNS disorders, such as ALS (amyotrophic lateral sclerosis), Parkinson’s disease and Alzheimer’s disease. Despite the critical importance of astrocytes in neurodegeneration and neuroinflammation are recognized, the lack of suitable *in vitro* systems limits their availability for modeling human brain pathologies. Here, we report the time-efficient, reproducible generation of astrocytes from human induced pluripotent stem cells (hiPSCs). Our hiPSC-derived astrocytes expressed characteristic astrocyte markers, such as GFAP, S100b, ALDH1L1 and AQP4. Furthermore, hiPSC-derived astrocytes displayed spontaneous calcium transients and responded to inflammatory stimuli by the secretion of type A1 and type A2 astrocyte-related cytokines.

## Introduction

Human induced pluripotent stem cells (hiPSCs) have revolutionized the field of human disease research, specifically in the areas of neurodegenerative disorders [1]. These cells have opened up new avenues for studying the underlying mechanisms of disease risk and onset in otherwise inaccessible patient-specific cells [2, 3]. In addition to disease modeling and mechanistic studies, hiPSC-based cellular models have a broad range of applications in the field of neuroscience, most importantly in highly relevant areas of neuronal drug development, target identification and molecular library screening, drug candidate efficacy and neurotoxicity testing, neuronal biomarker discovery, understanding inter-individual variability in neuronal and glia cells and networks activity, and individual susceptibilities. For example, hiPSC-derived cells can be used to assess the safety and efficacy of drugs in a human context in various stages of clinical trials and prior to their introduction into the market [4, 5]. Furthermore, hiPSC-based models can be used to develop personalized medicine, where patient-specific hiPSCs are used to generate cells to test the efficacy of different drugs for a particular patient or patient cohort [5]. Finally, hiPSC-based models can be used to study the effects of environmental toxins on human cells, which can help identify potential environmental and chemical hazards and develop strategies to mitigate their effects [6, 7].

Advances in hiPSC-based brain models paved the way for understanding the pathomechanisms of neurodegenerative disorders (amyotrophic lateral sclerosis (ALS) [8], frontotemporal dementia (FTD) [9], Huntington’s disease [10], Alzheimer’s [11] and Parkinson’s disease [12]) on the molecular and cellular levels. These *in vitro* neural models highlighted the significant contribution of activated glial cells to the exacerbation of neuroinflammation and subsequent neuronal damage. Hence, an enhanced focus emerged on the *in vitro* production of hiPSC-derived glial cells, such as the astrocytes.

Astrocytes are the most abundant cell types in the CNS with a remarkable heterogeneity both in morphology and function. The multifaceted functions of astrocytes highlight their critical role in maintaining the homeostasis and functionality of the nervous system, including structural support, immune response, neurovascular coupling, neurotransmitter uptake and recycling, ion and pH regulation [9, 13]. This kind of diversity in both morphology and physiological role may be the reason for the lack of availability of optimized short and cost-effective protocols to produce astrocytes from hiPSCs *in vitro*.

Changes in astrocyte morphology, transcriptional profile, and function, the hallmarks of astrocyte reactivation are observed in many neurodegenerative diseases, such as Alzheimer’s disease, Parkinson’s disease, Huntington’s disease, and ALS. Investigation of *in vitro* disease phenotype of astrocytes would greatly improve our understanding of disease pathomechanisms. Many initial events which causes changes in astrocyte morphology can be monitored by *in vitro* model system (hypertrophy [14], hyperplasia [15], process extension and ramification [16], reactive astrogliosis [17], scar formation [18], migratory changes [19], etc.).

A significant portion of hiPSC-derived astrocytic protocols supports differentiation with commercially available, undefined media [20]. Guides containing 100 days of cultivation show cells with similar expression profiles as *in vivo* primary astrocytes. Commercially available kits with defined medium provide faster generation of astrocytes than most of the traditional methods, though, their efficiency in functional astrocyte generation is documented only by a limited number of publications [21]. Furthermore, most of the defined astrocyte differentiation media use fetal bovine serum (FBS), which interferes with astrocyte-specific gene expression [22, 23]. FBS stimulates the proliferation of glial progenitor cells but imposes long-term changes in astrocyte’s gene expression decreasing their similarity to the *in vivo* astrocyte phenotypes [23]. The serum-based methods can provide fast generation, but incomplete maturation state of astrocyte-like cells since the use of serum disrupts astrocyte-specific gene expression and shifts the cell culture towards distinct phenotypes [23].

In this study, we aimed to develop an efficient, cost-effective method for the generation of astrocyte cultures with high purity from human induced pluripotent stem cell (hiPSC)-derived neural progenitor cells (NPCs). The implemented protocol has the advantage of significantly shortening the cultivation time starting from NPCs. The presented results show that the cells express the markers specific to astrocytes, in addition to the absence of neuronal markers that would indicate neuronal directional commitment. Moreover, the generated astrocytes proved to be functional in terms of cytokine secretion upon inflammatory stimuli, and they were able to propagate waves of calcium transients, which is a remarkable characteristic of astrocytes.

## Materials and methods

### Ethics statement

The ethical license was issued by the Scientific and Research Ethics Committee of the Hungarian Health Science Council for “Production of induced pluripotent stem cells (IPS) from human somatic samples” with the following ID No.: IV/3935–1/2021/EKU in May 2021.

### Materials and reagents

The chemicals, reagents and plasticware were purchased from Sigma-Aldrich (St. Louis, MO, USA), and Thermo Fisher Scientific (Waltham, MA, USA), unless specified otherwise.

### Cell lines

Two non-diseased human iPSC lines (BIOT009 and BIOT021) were used in this study, derived from healthy female donors and characterized earlier [24, 25]. Neural progenitor cells (NPC) were generated from the iPSCs by dual inhibition of the SMAD signaling pathway using LDN193189 and SB431542 [26, 27], then subsequently used for astrocyte differentiation.

### *In vitro* astrocyte differentiation

We generated astrocytes using our *in vitro* method available at protcols.io (DOI: dx.doi.org/10.17504/protocols.io.x54v9pb4mg3e/v1). We used previously established, characterized banks of NPCs generated by dual-SMAD inhibition method [26, 27]. Briefly, to generate astrocyte from the NPCs, we seeded 25.000–75.000 NPCs/cm^2^ into Matrigel-coated 6-well plates, and the medium was switched next day to astrocyte induction medium (AIM) (astrocyte growth medium (AGM, ScienCell) supplemented with 1X Astrocyte Growth Supplement (AGS, ScienCell), 2% FBS and 1% Pen/Strep). Cells were maintained in AIM for 21 days. Medium was changed every other day, and the cells were passaged using accutase upon reaching confluence. On Day 21, astrocyte progenitor cells were plated into Matrigel-coated plates (25.000–75.000/cm^2^) in serum-free astrocyte maturation medium (AMM), which was AGM supplemented with 1x AGS, 1% Pen/Strep and 20 ng/mL recombinant human ciliary neurotrophic factor (CNTF, provided by Peprotech). Cells were maintained in AMM for 21 more days with medium changes every other day, and passaged with TripLE express upon reaching confluence.

### RNA-sequencing and transcriptomic analysis

Total RNA from cellular pellet samples was isolated using TRI Reagent (Merck, USA) with RapidOutput DNA removal kit (ThermoFisher Scientific, USA). The quantity and quality of RNA extracts were analyzed using a Qubit 4 fluorometer (ThermoFisher Scientific, USA) and Fragment Analyzer (Agilent, USA). Complementary DNA (cDNA) libraries were synthesized using the NEBNext® Poly(A) mRNA Magnetic Isolation Module and NEBNext® Ultra™ II RNA Library Prep Kit for Illumina® (NEB, UK). The concentration and quality of cDNA libraries were evaluated with a Qubit 4 fluorometer (ThermoFisher Scientific, USA) and Fragment Analyzer (Agilent, USA), which was followed by the dilution of the cDNA samples to 4 nM. RNA sequencing was performed on the Illumina NovaSeq 6000 platform with NovaSeq S4 300 kit generated 150 bp pair-end reads (Illumina, USA). The raw sequencing data with corresponding metadata are available in the NCBI Gene Expression Omnibus (GEO) repository under accession number **GSE253372**.

The sequencing quality of each read was assessed by FastQC, erroneous reads were filtered out using Trim Galore. Trimmed sequence reads were mapped to the human genome sequence hg38 by using the R package Rbowtie2 [28] and Rsamtools (https://bioconductor.org/packages/Rsamtools). Differentially expressed genes (DEGs) with |log2 fold change| > 1 and q < 0.01 (FDR-adjusted P-values) were selected in DeSeq2 [29]. To cluster the sorted DEGs, we used the R packages pvclust and Dendextend [30]. Heatmaps were constructed using the R package ComplexHeatmap [31]. Cellular deconvolution was carried out using BrainDeconvShiny [32] with the assistance of dtangle [33] and transcriptomic data from the primary astrocytes [34].

### Immunocytochemistry (ICC)

25.000–75.000 astrocytes/cm^2^ were plated into Matrigel-coated 24-well plates, (containing glass coverslips). On the next day, the cells were fixed with 4% paraformaldehyde, which was followed by permeabilization with 0.2% Triton X-100. Samples were blocked by 3% BSA for 1 hour and incubated with the primary antibodies indicated in S1 Table overnight. Next day, the samples were washed with phosphate-buffered saline (PBS), then incubated with secondary antibodies goat anti-chicken Alexa-488 (#A11039), donkey anti-rabbit Alexa-599 (#A21207) and donkey anti-mouse Alexa-647 (#A31571), (all purchased from Invitrogen). Nuclei were stained with DAPI. ImageXPress NANO with the MetaXPress software (Molecular Devices) was used for high-content imaging. Negative control stainings were performed for both cell lines by using all secondary antibodies, no unspecific staining was detected (S2 Fig).

### Western blotting (WB)

Samples for Western blot analysis were harvested at various stages of astrocyte differentiation. Cells were washed in 1X PBS, then RIPA lysis buffer was added and monolayers were removed by scraping. Samples were incubated in lysis buffer for 30 minutes and then centrifuged at 13,000 rpm at 4°C for 20 minutes. Supernatants were collected and stored at -80°C. Protein concentration was measured using Pierce™ BCA Protein Assay Kits (ThermoFisher Scientific, USA) and equal amounts of total protein (50 μg per lane) were separated by SDS-polyacrylamide gel electrophoresis in mPAGE™ 4–20% Bis-Tris Precast Gels (Merck Millipore, USA). Proteins were transferred to Immobilon-E membrane (Merck Millipore, USA), blocked with 5% BSA in 1X Tris-buffered saline (TBS buffer) and incubated overnight at 4°C with the primary antibody in TBS-T (TBS with 0.1% TWEEN-20) buffer containing 5% BSA (S2 Table). After three washes with TBS-T buffer, the membranes were incubated with secondary antibodies (S2 Table) and visualized using Immobilon Crescendo Western HRP Substrate (Merck Millipore, USA) and Fusion FX system (Vilber, France). The original uncropped blots are presented in S1 Fig.

### Cytokine profiling

As part of the functional characterization, astrocyte cultures (Day 42) were incubated with 10 ng/mL IL-1β (PeproTech) + 10 ng/mL TNF-α (PeproTech) for 24 hours, then supernatant samples were collected from control and stimulated cultures to analyze the secreted cytokines by using Proteome Profiler Human XL Cytokine Array kit (R&D, ARY022). The applied array is capable of detecting 105 soluble human proteins (multiple cytokines, chemokines, growth factors and other soluble proteins, listed in S3 Table). The collected supernatant samples were incubated overnight on nitrocellulose membranes spotted with capture and control antibodies, then the membranes were washed to remove unbound material, followed by incubation with a cocktail of biotinylated detection antibodies. Streptavidin-HRP and chemiluminescent detection reagents were then added, and Kodak Gel Logic 1500 Imaging System was used to document the results. The signal produced at each capture spot corresponds to the amount of protein bound, which was quantified using GelQuant.NET software provided by biochemlabsolutions.com.

### Time-lapse calcium imaging

Spontaneous intracellular calcium transients were measured in astroprogenitor cells (Day 21) and astrocytes (Day 42) loaded with Fluo-4 Direct fluorescent calcium indicator dye in the presence of Probenecid organic anion transporter (Thermo Fisher Scientific, F10471). The medium was removed from the cultures and after the addition of 5x diluted Fluo-4 Direct loading solution, the cells were incubated for 60 minutes at 37°C protected from light. Live cell fluorescent imaging and time-lapse microscopic measurements were performed using an ImageXpress Nano Automated Imaging System (Molecular Devices) controlled by the MetaXpress software. A standard fluorescein isothiocyanate (FITC) filter was used with 20x and 40x objectives, and images were acquired every second for 5 min. Excitation light intensity was 100% with an exposure time of 2–5 msec. Three sites were imaged for each sample and the signals obtained were analyzed using the calcium signal analyzer software CaSiAn [35]. Time-lapse movies were created with the MetaXpress software, which are included as (S1–S6 Movies).

## Results

### *In vitro* characterization of induced astrocyte differentiation

**Fig 1:**
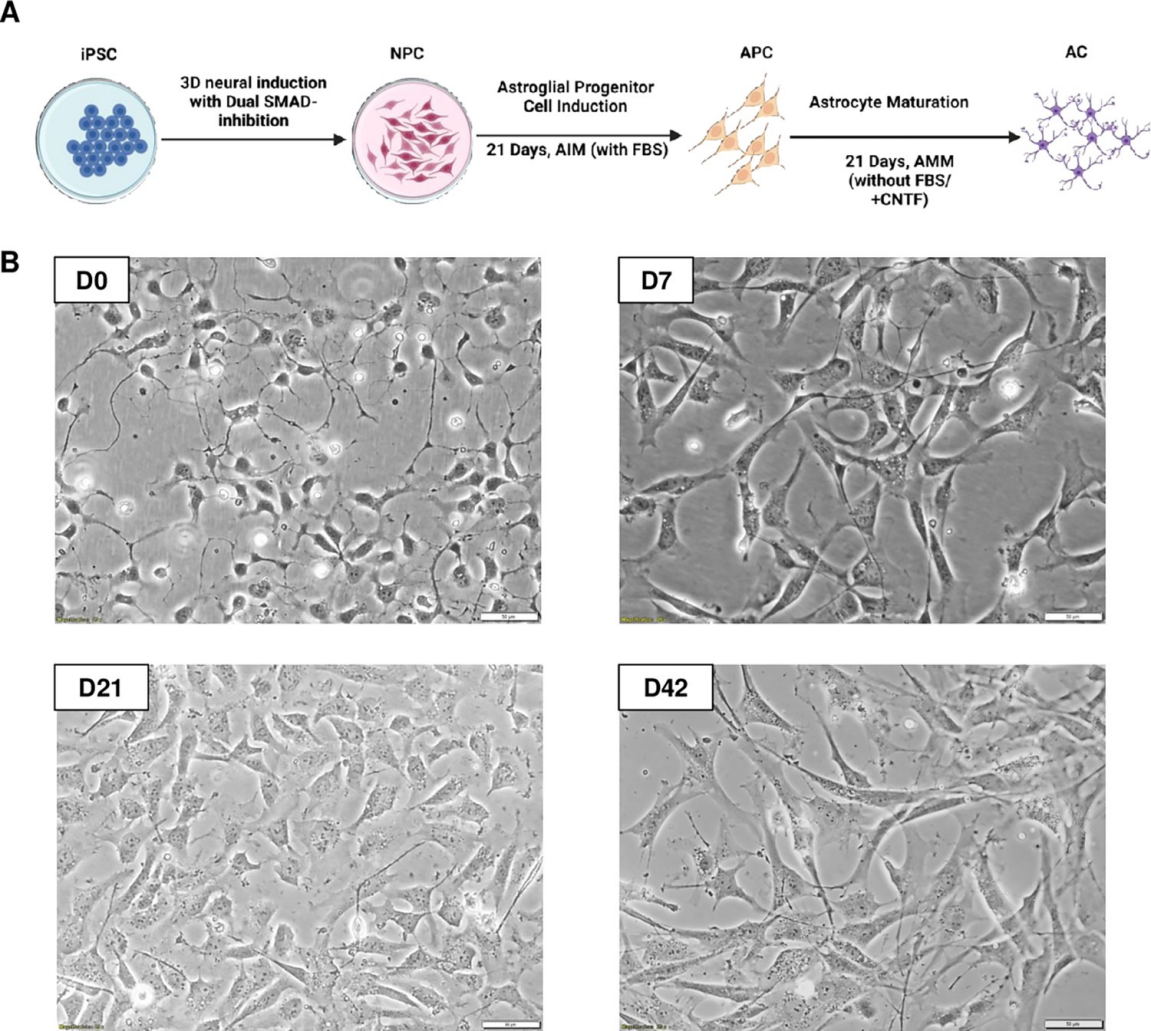
Representative cell morphology changes during astrocytes differentiation. (A) Overview of the *in vitro* method used for astrocytes generation from hiPSC-derived NPCs. (B) Light microscopic images were taken before initiating astrocyte induction (Day 0), Day 7 of astrocyte induction, Day 21 of astrocyte induction and Day 42 of astrocyte maturation. (Scalebar: 50 μm).

We generated astrocytes from previously established cell banks of hiPSC-derived NPCs obtained by the dual-SMAD inhibition method. Our *in vitro* astroglial differentiation protocol is based on the modified version of the method published by TCW et al (Fig 1A). Although FBS boosts the proliferation of astroglial progenitor cells, it was reported, that prolonged use of serum perturbs the expression of mature astrocyte-specific genes. In addition, the permanent use of FBS throughout the entire period of differentiation exerts an adverse effect on astrocyte-specific gene expression and triggers the generation reactive astrocyte phenotypes [23, 36]. Taken these findings into consideration, we provided (2%) FBS only for the first three weeks of astrocytes differentiation to allow the enrichment of astroglial progenitor cells (APCs). For the next three weeks of astrocyte maturation, we used serum-free medium. Although glial specification is initiated by the coordinated action of transcription factors nuclear factor Ia (NFIA) and SRY-Box Transcription Factor 9 (Sox9) [37], astroglial differentiation and astrocyte- specific gene expression requires the synergistic activation of the SMAD and STAT3 transcription factors via tyrosine-kinase (BMP4) and type I cytokine-receptors (CNTF, LIF). As CNTF is a potent activator of JAK-STAT and MAPK pathways [38], we included it in the astrocyte maturation medium in a concentration of 20 ng/mL.

The morphology of the cells profoundly changed during *in vitro* astrocyte differentiation (Fig 1B). The initial NPC culture displayed spindle-shaped cell-morphology and colonies forming neural rosettes with some neurites in the cell culture. As the astroglial differentiation progressed the cells acquired an elongated, triangular shape, and the neurites were vanished by Day 21. From Day 21, more processes emerged from the somata giving rise to star-shaped cells in the astrocyte culture. Another hallmark of astrocyte differentiation, the increased granularity of the cell bodies was also observed, which reflects the increased secretory activity of astrocytes and is connected to increased protein synthesis, glycogen accumulation, lysosomal activation, and the storage of lipids or metabolites as well [39].

**Fig 2:**
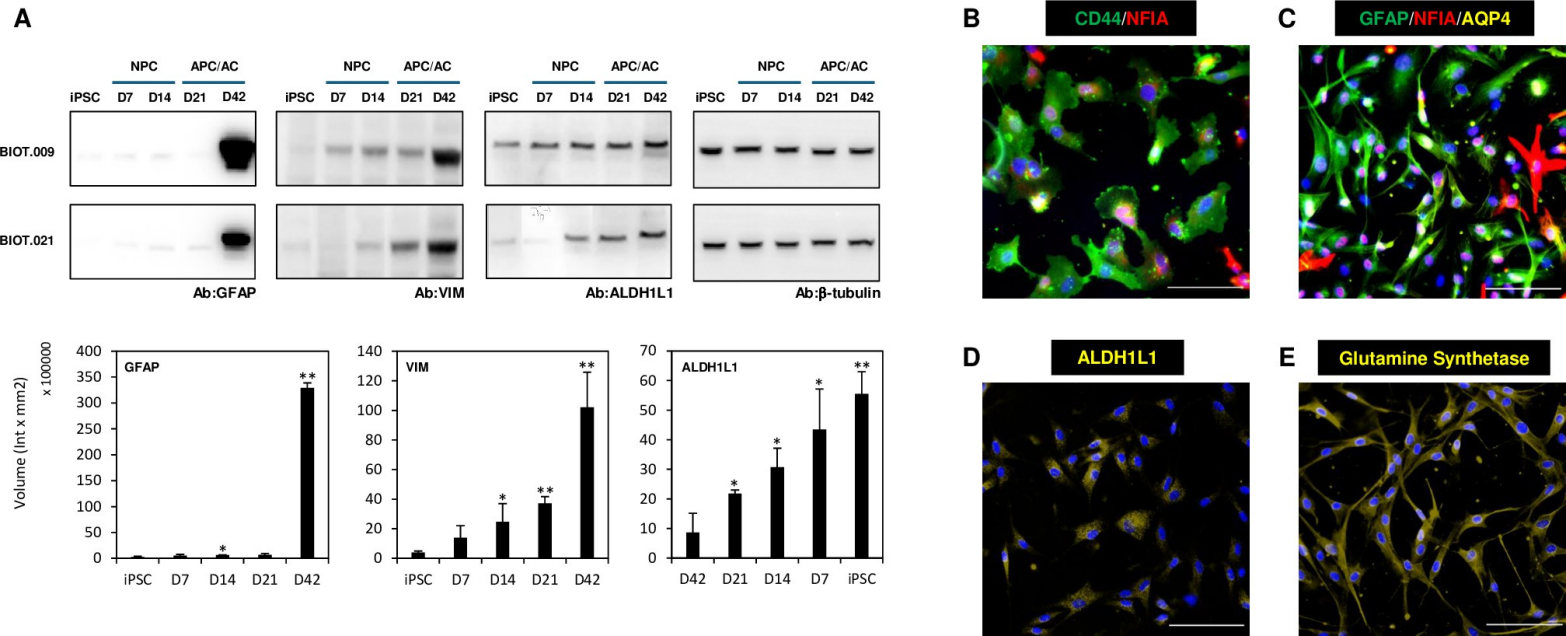
The expression of astrocytic markers. (A) Western blotting (WB) was used to analyze the typical astrocytic marker protein expression (GFAP, VIM, and ALDHL1) during the differentiation of astrocytes from iPSCs. After protein separation by SDS-PAGE, the proteins were blotted onto a PVDF membrane for antibody detection. Chemiluminescence detection was used to visualize the labeled bands using β-tubulin as a loading control. The average intensities of the signals ± SD were calculated from WB images of two independent experiments with two individual cell lines (BIOT.009 and BIOT.021) and compared to iPSC (Student’s t-test, *p < 0.05, ** p < 0.01, n = 4). The original uncropped blots are presented in S1 Fig. (B) Representative images show the expression of AP markers CD44 (green) and NFIA (red), detected by immunocytochemistry. (Scalebar: 100 μm). (C) Representative immunocytochemical staining of GFAP (green), NFIA (red) and AQP4 (yellow) in Day 42 astrocytes. On Day 42, the expression of astrocyte markers ALDH1L1 (D) and GS (E) were also detected (yellow, scalebar: 100 μm). DAPI was used as a nuclear counterstain.

We observed a progressive change in the cellular marker expression during *in vitro* astrocyte generation. Day 21 APCs expressed CD44 and nuclear factor IA (NFIA) (Fig 2B), which are characteristic markers of astrocyte progenitor cells. Day 21 APCs were also analyzed for mature astrocyte markers expression, which were stained together with vimentin (VIM), glial fibrillary acidic protein (GFAP) and NFIA (S3 Fig). APCs displayed mostly faint expression of the mature astrocyte markers (aquaporin 4 (AQP), aldehyde dehydrogenase 1 family member L1 (ALDH1L1), glutamine synthetase (GS)). On the other hand, Day 42 astrocytes were highly positive for GFAP and classical astrocyte markers such as S100 calcium binding protein B (S100β), VIM, ALDH1L1, GS and AQP4, as shown by ICC (Fig 2C–2E) and WB (Fig 2A). We observed moderate astroprogenitor marker expression (CD44, NFIA) in astrocyte cultures on Day 42 (S4 Fig), as reported in other studies investigating astrocyte development [40–42]. Thus, the molecular phenotype of the cells suggests the *in vitro* recapitulation of astrocyte development throughout the intermediate astroglial progenitor stage. At this stage, cells express markers associated with astrocyte lineage commitment but have not yet acquired the morphological and functional characteristics of mature astrocytes.

### Transcriptomical analysis reveals an astrocyte-specific signature

Astrocyte maturation is accompanied by the sequential repression of neuronal, microglial and oligodendrocyte lineage genes, and subsequent activation of astrocyte-specific genes [43]. Transcriptomic analysis performed on differentiating astrocytes indicates the emergence of an immature astrocyte phenotype, which is characterized by the upregulation of the canonical astrocyte markers, but lacks several genes involved in astrocyte-specific functions, such as glutamate homeostasis and calcium signaling. Further maturation provides the transition from this intermediate astrocyte stage into the functionally mature astrocyte phenotype [22, 43–45].

**Fig 3:**
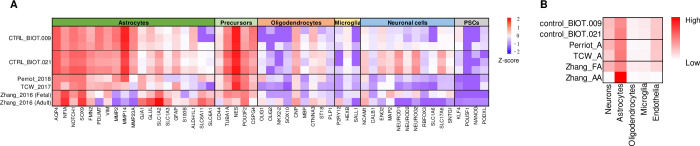
The degree of differentiation of the astrocytes derived from hiPSCs. (A) Heatmap showing basemean expression profile of the 52 marker genes from hiPSCs-derived astrocytes from the study of Perriot et al. 2018 [23] and TCW et al. 2017 [20] and fetal astrocytes from the study of Zhang et al. 2016 [34]. The 52 markers are listed on the x-axis, and hiPSCs-derived astrocytes on the y-axis. Results are expressed as the Z-score of the log2 basemean values. (B) Heatmap showing the percentage of generated astrocytic cultures sharing the expression signatures with the human adult brain tissue extracted during surgery [34]. Cellular deconvolution was carried out using BrainDeconvShiny [32] with the assistance of dtangle [33].

To assess the maturity of the astrocyte cultures, we conducted RNA sequencing using bulk RNA-seq platforms. Our analysis revealed elevated expression of astrocyte-specific genes, such as *GFAP*, *AQP4*, *NFIA*, *SOX9*, and *SLC1A2*. Furthermore, we observed high expression level of genes involved in the organization of the extracellular matrix (ECM) process, including *MMP2*, *MMP14*, and *MMP23A*. This finding is consistent with the important role of astrocytes in producing ECM molecules [46]. In contrast, most neuronal genes (*RBFOX3*), oligodendrocyte genes (*SOX10*, *OLIG-2*, *NKX2*.*2*), microglial genes (*P2RY12*, *SALL1*), and pluripotency-related genes (*KLF4*, *POU5F1*, *NANOG*, *PODXL*) exhibited low gene expression (Fig 3). The gene expression pattern was highly similar to the gene activation pattern previously reported in fetal astrocytes [34]. Furthermore, the astrocyte culture was characterized using bulk RNA-seq platforms with the ’dtangle’ deconvolution method [33]. The proportion of cell types present in our astrocyte culture was estimated in comparison to fetal and primary astrocytes from a previous study [34], as well as to hiPSCs [17, 20], using transcriptomic data obtained from primary and fetal astrocytes [34]. Except for primary astrocytes, we were unable to identify a cell culture with an entirely matching gene expression profile (Fig 3B). The astrocytes that were differentiated using our method exhibited a distribution that was closely similar to that of the previous methods [17, 20] and fetal astrocytes (Fig 3B). This indicates that our astrocyte cultures exhibit characteristics of the intermediate/immature astrocyte phenotype.

### hiPSC-derived astrocytes respond to pro-inflammatory stimuli

**Fig 4:**
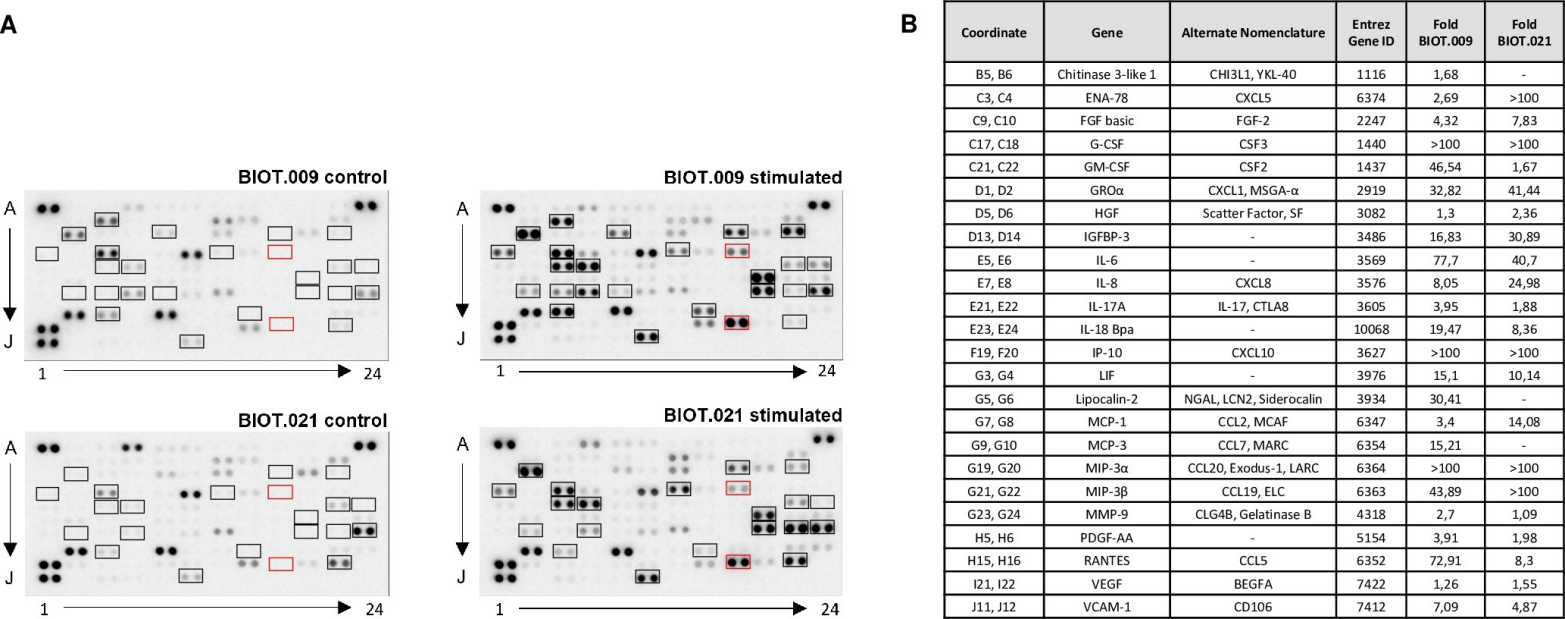
Cytokine/chemokine profiling. (A) The applied array is suitable for detecting 105 human cytokines, chemokines, growth factors and other soluble proteins (S3 Table). Left panels show their secretion from control, non-stimulated astrocyte cultures, right panels show the secretion from astrocyte cultures stimulated with IL-1β and TNF-α. Framed spots in red indicate the stimulator molecules (IL-1β and TNF-α) and the spots with black frame indicate the cytokines that were secreted in higher amounts upon stimulation. (B) List of the top-24 cytokines/chemokines whose secretion levels were altered following IL-1β+TNFα treatment. Spot signal intensity was quantified using GelQuant.NET software provided by biochemlabsolutions.com. Newly expressed molecules that were not detectable in the cell culture supernatant samples without treatment are indicated with > 100-fold change. IL-1β and TNF-α used for pro-inflammatory stimulation were not listed.

Astrocytes produce cytokines and chemokines to regulate many neuronal functions *in vivo*, with both neurotoxic (inflammatory) and neuroprotective (immune-regulatory) roles in the brain. Cultured human astrocytes also express a distinct set of cytokines and chemokines in resting and activated conditions [47]. To further characterize our astrocytes and to test whether they respond to pro-inflammatory stimuli, cells were treated with IL-1β and TNF-α for 24 hours, and then cell culture supernatant was collected from control and stimulated cultures to analyze the secreted cytokine profile using a protein microarray. We found that following 24 hours of exposure to a mixture of IL-1β and TNF-α, the secretion levels of several cytokines/chemokines including GROα, IGFBP-3, IL-6, IL-8, RANTES and VCAM-1 were substantially increased in both cell lines (Fig 4). In addition, we could also detect newly produced molecules after the stimulation, such as G-CSF, IP-10, MIP-3α and MIP-3β, indicating the functionality of the astrocytes generated by the applied protocol. Intriguingly, the matrix metalloproteinase-9 (MMP-9), which is expressed in astrocytes [48] and implicated in CNS tissue remodeling as well as in immune cell infiltration, was also upregulated.

### hiPSC-derived astrocytes conduct calcium waves

Another pivotal function of astrocytes is the generation of calcium waves, which govern signal transduction, the synthesis of bioactive molecules, gliotransmission and cytokine secretory pathways. Astrocyte calcium waves are not only evoked in response to receptor activation by neurotransmitter molecules (ATP, GABA and Glutamate) but are also triggered spontaneously. Calcium fluxes through calcium permeable channels, sodium-calcium exchangers and intracellular calcium stores (endoplasmic reticulum, mitochondria) promote spontaneous calcium fluctuations, which are characterized by slow spreading kinetics, usually with an interspike interval of 100–150 sec.

**Fig 5:**
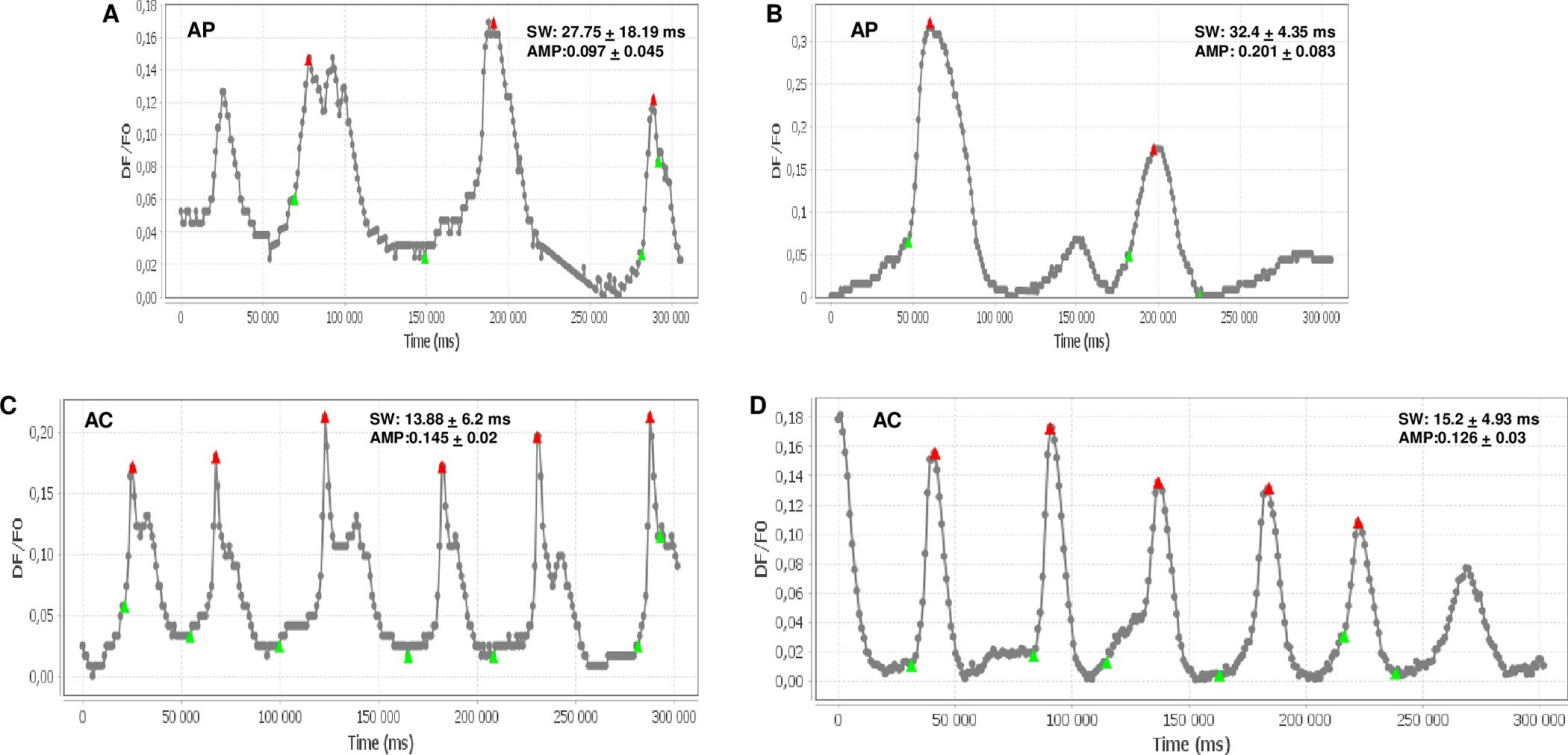
Day 21 astroglial progenitor cells and Day 42 astrocytes conduct calcium waves. Astroglial progenitor cells (AP) at Day 21 and astrocytes (ACs) at Day 42 were loaded with the calcium-sensitive dye Fluo-4. Fluorescence intensity changes were recorded by time-lapse imaging (1 image/second for 5 minutes). Curves representing fluorescence change (DF/F0) over time—with signal width (SW) and amplitude (AMP) values—were obtained in CaSian. APs from the BIOT.009 (A) and BIOT.021 (B) cell lines displayed slow spikes with high SWs, whereas the maturated astrocytes from BIOT.009 (C) and BIOT.021 (D) lines produced rhythmic calcium waves with higher frequencies.

To assay calcium fluctuations in our *in vitro* generated astrocytes, we performed Fluo-4 labeling combined with efflux inhibitor molecule probenecid. Fluctuations in intracellular calcium levels were measured by timelapse imaging as described above. We detected several repeatedly activated regions in the measurements, for which the curves were extracted and analyzed (Fig 5, S1–S6 Movies). These curves showed repeated calcium spikes with an average interspike interval of around 50 seconds in astrocytes. Although the calcium spikes were also detected in Day 21 APs, they appeared with slower kinetics and lower frequencies (Fig 5A and 5B) compared to the calcium spikes detected in Day 42 astrocytes (Fig 5C and 5D). These results underpin the ability of our astrocytes to generate spontaneous calcium waves.

## Discussion

Although several approaches were published for *in vitro* astrocyte generation [49–52], the availability of efficient, time- and cost-effective methods for the differentiation of human astrocytes is still limited. The main limitations of the current methods are the extremely long differentiation periods (varying between 70 and 150 days) [53, 54], the high qualitative and quantitative variability in the applied reagents and the lack of comprehensive characterizations. Here, we report the differentiation of functional astroglial cells from human iPSC-derived NPCs within 42 days, which produce calcium waves and respond to inflammatory stimuli by secretion of type A1 and type A2 cytokines.

Early passage NPCs are multipotent stem cells, and they have the potential to generate both neuronal and glial lineages. Here, we used our previously established control NPC banks allowing us to generate astrocytes without the need for neural induction. Furthermore, protocols for cryopreservation of glial progenitors and astrocytes were also published [55, 56]. With pre-existing NPC banks, our differentiation method can generate functional astrocytes within 42 days, although, with established astroglial progenitor cell banks, this differentiation period can be further shortened.

Replacing the lengthy astrocyte differentiation methods with cost-effective, rapid differentiation protocols is a common endeavour of the field of astroglial research (Table 1). Although several novel approaches were published [20, 36, 57], their impact on the reactivity states and gene expression profile of the mature astrocyte culture must be taken into consideration. For example, it was reported, that the use of FBS for the entire period of astrocyte differentiation profoundly rewires gene expression and generates reactive astrocyte phenotypes [22, 23, 58]. Likewise, the use of AraC to produce quiescent, mature astrocytes carries the risk of rendering them to a neurotoxic, A1-like phenotype [59]. Genetic methods using viral vectors offer a rapid transcriptional-programing driving cells towards astroglial differentiation [57, 60–62]. Transient activation or overexpression of NFIA, SOX9 and NGN2 led to rapid acquisition of astrocyte-specific morphologies, markers, and functions in cell cultures. However, these methods are limited by high cost and time demands of viral vector production, highly variable transfection efficiencies, and high variabilities in cell viability upon transfection. Furthermore, there are concerns about random vector integration (location of the insertion site, multiple copies of the genome-integrated vector), which may result in inadequate expression patterns and/or overexpression artefacts in cell characteristics [63, 64].

**Table 1:** Methods published for rapid (≤ 60 days) *in vitro* generation of astrocytes from stem cell-derived NPCs.

Reference	Length of Differentiation (days, from NPC stage)	Markers	Assays	Factors/Supplements used
**TCW et al, 2017 [20]**	30 days	ALDH1L1, APOE, GLAST, ACSBG1, AQP4, S100β, VIM	IL-6 secretion, (spontaneous/evoked) calcium-imaging, phagocytosis	AGS with 2% FBS
**Voulgaris et al, 2022 [50]**	28 days	CD44, GFAP, AQP4, S100β, ALDH1L1, VIM, EAAT1, EAAT2	Glutathione assay, Il-1β, IL-6 and IL-8 secretion, glutamate uptake	2% FBS, bFGF
**Sherida M. de Leeuw et al, 2022 [36]**	31 days	GJA1, S100β, IQGAP1, S100A6	Cytokine secretion, lysosomal cholesterol enrichment, Aβ uptake	10% FCS (induction), araC (maturation)
**Magistri et al, 2016 [22]**	7 days	GFAP, VIM, AQP4, CD44	*in vitro* characterization only	2.5% FBS or 20 ng/mL CNTF/BMP4 (in DMEM/F12 +N2)
**Mulica et al, 2023 [58]**	Long, serum-free (LSF) method: 140 days	VIM, GFAP	*in vitro* characterization only	LSF: 10 ng/mL BFGF, 10 ng/mL EGF, 10 ng/mL CNTF, 10ng/mL BMP4 SSC: 1%FBS
	Short, serum-containing (SSC) method: 60–67 days			

Despite GFAP being an important and widely used marker for the identification of astrocytes, its expression is highly variable between different astrocyte cultures, different brain regions, and between distinct subgroups of astrocytes [65]. GFAP expression may vary in response to changes in environmental or cell culture conditions (e.g. passage number [66]), changes in media components especially growth factors, and has high donor-to-donor variability. In addition, GFAP is also expressed by early glial progenitor cells, which generate both astrocytes and oligodendrocytes [67]. On the other hand, ALDH1L1, APQ4, GS and solute carrier family 1 member 2 (SLC1A2) are bona fide astrocyte markers, and routinely used for *in vitro* characterization of human iPSC-derived astrocytes [55]. In addition, a set of extra markers (Ror, Lhx2, Fezf2, Dbx2) were identified, which distinguish mature, adult astroglial cells from immature astrocytes.

Maturation of astroglial progenitor cells is a critical step of *in vitro* astrocyte differentiation methods, and its efficiency depends on the soluble factors used, the length and timing of the maturation period. Studies using single-cell RNA-sequencing revealed an intermediate subtype of astrocyte lineage cells emerging during maturation. Cells in this maturation stage, termed „immature postmitotic astrocytes” display the expression of classical astrocyte markers, but they do not perform all mature astrocyte functions [43, 68, 69]. Immature astrocytes express the characteristic astrocyte markers GFAP, ALDH1L1, S100β, AQP4 and GS, although they acquire branching morphology and the expression of adult astrocyte markers (Lhx2, Dbx2, Rorb, FezF2). Due to their morphological traits and the expression of canonical astrocyte markers detected in our cells, phenotypes of our astrocyte culture resemble immature astrocytes. The use of a prolonged period of serum-free astrocyte maturation has the potential to enhance the maturation state of astrocytes. Although, astrocytes grown in long-term culture are prone to undergo replicative senescence [70]. In addition, the loss of physiological functions, and gain of reactive/neurotoxic phenotypes in senescent astrocytes were reported in long term cultures [71, 72]. Nevertheless, we obtained astrocytic cells after 42 days of differentiation, which were able to perform characteristic physiological astrocytic functions, such as calcium-wave conduction and cytokine secretion.

Our *in vitro* differentiation protocol produced APC-like intermediate cells by Day 21, which were positive for astroglial progenitor markers NFIA and CD44 (Fig 2B). NFIA is a master regulator of astrocyte-specific gene activation [62], and its upregulation indicates the commitment of our cells towards the astroglial lineage. By the 42nd day of differentiation, polygonal-shaped APCs gave rise to stellar-shaped cells with characteristic astrocyte morphologies (Fig 1B). Furthermore, the expression of GFAP, VIM, AQP4, GS and ALDH1L1 were confirmed in these cells by western blotting (Fig 2A) and ICC (Fig 2B–2E). Transcriptome analysis revealed the upregulation of additional astrocyte-specific genes (*NOTCH1*, *SOX9*, *FMN2*, *GJA1*) besides the downregulation of several genes from the microglial, neuronal, and oligodendrocyte lineages (Fig 3). The obtained gene expression profiles of our astrocytes differentiated from the two hiPSC lines showed high similarity to those published for astrocytes generated from hiPSC-derived NPCs [20, 23]. These results corroborate the astrocytic phenotype of our *in vitro* differentiated cell lines.

In addition to the assessment of the molecular phenotypes, we showed, that our differentiated cells perform key astrocyte-specific functions, such as the production of spontaneous calcium waves and secretion of cytokines in response to inflammatory stimuli. Calcium waves in astrocytes are typically associated with a subtype known as "interlaminar astrocytes" or "process-bearing astrocytes" [73]. These astrocytes have long processes and are found in the gray matter of the brain. They are often referred to as "complex" or "non-territorial" astrocytes due to their intricate morphology, which includes numerous fine processes that extend into the neuropil, making contact with synapses and blood vessels. The phenomenon of calcium waves in astrocytes involves changes in intracellular calcium concentrations, and it is considered a form of intercellular communication within astrocytic networks. When one astrocyte experiences a calcium transient, it can propagate to neighboring astrocytes, leading to a wave-like spread of calcium signals [74]. This form of communication is known as "astrocytic calcium signaling" or "gliotransmission” [75]. Calcium waves are triggered by neurotransmitter molecules (ATP, GABA, glutamate) binding to G-protein coupled receptors expressed in astrocytes. Intracellular and intercellular spreading of the calcium wave is maintained by various mechanisms around the soma and astrocyte processes, such as intracellular calcium stores (ER, mitochondria), Na/Ca2+ exchangers, Calcium-permeable channels and gap junctions. On the other hand, astrocytes conduct spontaneous, rhythmic calcium waves in the absence of external stimuli, which is mediated by natrium/calcium exchange in astrocytic processes and maintained by calcium-induced calcium release through IP3R activation [76, 77]. In contrast to transmitter-evoked calcium waves, spontaneous calcium waves have lower amplitude, lower frequency and higher inter-spike intervals (100–400 sec) [73, 78]. By staining the cells with the calcium-sensitive Fluo-4, and utilizing time-lapse fluorescence imaging, we showed that our APCs and astrocytes generate spontaneous calcium spikes (Fig 5A–5D). Calcium spikes measured in APCs were much slower (with higher signal widths) than the repeated calcium oscillations observed in maturated astrocytes (Fig 5C and 5D). These results imply, that our *in vitro* method recapitulates the basal, spontaneous calcium dynamics of APCs and differentiated astroglial cells.

Astrocytes respond to CNS injury and inflammation by releasing numerous cytokines, which regulate blood-brain-barrier (BBB) permeability, microglial polarization and neuronal survival. The cytokine secretion profile of astrocytes is shaped by microenvironmental cues, such as the phenotypic composition of the surrounding microglia [79, 80]. In response to danger/pathogen-associated molecular patterns, activated microglia signal through cytokines (IL1-β, TNFα), which activate diverse subsets of reactive astrocytes. In, turn, reactive astrocytes modulate microglial responses through pro-inflammatory (IL1-β, RANTES, CXCL-2, G-CSF) or anti-inflammatory (IL-10, IL-6, TGFβ) mediators. To evoke inflammatory astrocyte-mediated cytokine responses, we used microglial-derived pro-inflammatory mediators, TNFα and IL1-β. Twenty-four hours of stimulation by TNFα and IL1-β triggered a massive inflammatory response from astrocytes (Fig 4), including the upregulation of microglia activating (IL-6, GM-CSF, GROα), BBB-regulating (VEGF, CCL-2, CXCL-10) and neuroinflammatory cytokines (IL-8, MMP9, ENA-78, Serpin E1). Similar results were obtained in other studies [47, 55, 81–83], with more pronounced upregulation of other soluble messengers, such as RANTES, MIP-1 and CXCL-5. Of note, the differences observed in the literature might stem from multiple sources, such as the differences in the incubation times (24 hours versus 7 days), the applied inflammatory stimuli (C1q, IL1-α), the differentiation protocols and cell lines used for these experiments. Nevertheless, we evoked inflammatory responses in our cell cultures, which are characteristic of pro-inflammatory reactive astrocytes.

It is an important issue, how much the generated astrocytes can be identified as a specific known *in vivo* astrocyte subpopulation. In the central nervous system, astrocytes can be broadly classified into protoplasmic and fibrous types based on their morphology and location [84]. Protoplasmic astrocytes are found in gray matter and involved in synaptic regulation and have a complex, bushy morphology. They typically express markers such as GFAP and AQP4 [85]. Fibrous astrocytes are found in white matter, exhibiting a more elongated morphology and are involved in maintaining myelin. They may express markers like GFAP and VIM [86]. Faithful *in vitro* recapitulation of such phenotypes would be very valuable, but conceptually might be difficult, as the *in vivo* niche of multiple strongly interacting neuronal and glia cell types and extracellular matrices in a 3D structure would require a complexity and dynamic control of cell culture conditions not achieved by the current state-of-the-art methodologies [87]. Nevertheless, the “astrocyte only” culture and maturation system described in this paper provides important and well defined starting material to generate more complex systems and facilitate to decipher the specific cell to cell interactions and contributing factors in model systems.

Taken together, we presented the *in vitro* generation of functional astrocytes from human iPSC-derived NPCs through an intermediate astroglial progenitor cell stage within 42 days. In the future, evaluation of maturity, subpopulation heterogeneity and functionalities of astrocytes obtained by this method will be further extended by the utilization of other functional assays (glutamate uptake, phagocytosis), metabolomic analysis and single-cell RNA-sequencing.

## Conclusion

The protocol described in this paper have successfully resulted in functional astrocytes from human iPSCs, confirmed by relevant cellular markers and functional assays. The results suggest, that these *in vitro*-produced astrocytes can be used to study *in vitro* neuroglia toxicity and suitable for drug testing and modeling the pathomechanism of CNS diseases. Furthermore, they are appropriate starting materials for understanding the role and interactions of astrocytes in the human CNS.

## Supporting information

S1 Table Primary antibodies used for immunocytochemistry. (PDF)

S2 Table Antibodies used for Western blot. (PDF)

S3 Table List of controls and cytokines/chemokines analyzed, indicating their location on the Cytokine Array. (PDF)

S1 Fig Original uncropped Western blot images. (PDF)

S2 Fig Negative control immunostainings with the secondary antibodies used in this study. (PDF)

S3 Fig Expression of mature astrocyte markers in Day 21 astroglial progenitor cells. (PDF)

S4 Fig Expression of astroglial progenitor markers in Day 42 astrocyte cultures. (PDF)

S1 Movie BIOT.009 hiPSC-derived astroglial progenitor cells conduct calcium waves (20x objective). (AVI)

S2 Movie BIOT.021 hiPSC-derived astroglial progenitor cells conduct calcium waves (20x objective). (AVI)

S3 Movie BIOT.009 hiPSC-derived astrocytes conduct calcium waves (20x objective). (AVI)

S4 Movie BIOT.021 hiPSC-derived astrocytes conduct calcium waves (20x objective). (AVI)

S5 Movie BIOT.009 hiPSC-derived astrocytes conduct calcium waves (40x objective). (AVI)

S6 Movie BIOT.021 hiPSC-derived astrocytes conduct calcium waves (40x objective). (AVI)

## References

[1] Trudler D, Ghatak S, Lipton SA. Emerging hiPSC Models for Drug Discovery in Neurodegenerative Diseases. Int J Mol Sci. 2021;22(15). Epub 20210730. doi: 10.3390/ijms22158196 ; PubMed Central PMCID: PMC8347370. 34360966 PMC8347370

[2] Grekhnev DA, Kaznacheyeva EV, Vigont VA. Patient-Specific iPSCs-Based Models of Neurodegenerative Diseases: Focus on Aberrant Calcium Signaling. Int J Mol Sci. 2022;23(2). Epub 20220106. doi: 10.3390/ijms23020624 ; PubMed Central PMCID: PMC8776084. 35054808 PMC8776084

[3] Paik DT, Chandy M, Wu JC. Patient and Disease-Specific Induced Pluripotent Stem Cells for Discovery of Personalized Cardiovascular Drugs and Therapeutics. Pharmacol Rev. 2020;72(1):320–42. doi: 10.1124/pr.116.013003 ; PubMed Central PMCID: PMC6934989. 31871214 PMC6934989

[4] Elitt MS, Barbar L, Tesar PJ. Drug screening for human genetic diseases using iPSC models. Hum Mol Genet. 2018;27(R2):R89–R98. doi: 10.1093/hmg/ddy186 ; PubMed Central PMCID: PMC6061782. 29771306 PMC6061782

[5] Qu Y. hiPSC-Based Tissue Organoid Regeneration. sine loco: IntechOpen; 2018.

[6] Horanszky A, Shashikadze B, Elkhateib R, Lombardo SD, Lamberto F, Zana M, et al. Proteomics and disease network associations evaluation of environmentally relevant Bisphenol A concentrations in a human 3D neural stem cell model. Front Cell Dev Biol. 2023;11:1236243. Epub 20230816. doi: 10.3389/fcell.2023.1236243 ; PubMed Central PMCID: PMC10472293. 37664457 PMC10472293

[7] Lamberto F, Shashikadze B, Elkhateib R, Lombardo SD, Horanszky A, Balogh A, et al. Low-dose Bisphenol A exposure alters the functionality and cellular environment in a human cardiomyocyte model. Environ Pollut. 2023;335:122359. Epub 20230809. doi: 10.1016/j.envpol.2023.122359 . 37567409

[8] Du H, Huo Z, Chen Y, Zhao Z, Meng F, Wang X, et al. Induced Pluripotent Stem Cells and Their Applications in Amyotrophic Lateral Sclerosis. Cells. 2023;12(6). Epub 20230322. doi: 10.3390/cells12060971 ; PubMed Central PMCID: PMC10047679. 36980310 PMC10047679

[9] Chandrasekaran A, Dittlau KS, Corsi GI, Haukedal H, Doncheva NT, Ramakrishna S, et al. Astrocytic reactivity triggered by defective autophagy and metabolic failure causes neurotoxicity in frontotemporal dementia type 3. Stem Cell Reports. 2021;16(11):2736–51. Epub 20211021. doi: 10.1016/j.stemcr.2021.09.013 ; PubMed Central PMCID: PMC8581052. 34678206 PMC8581052

[10] Kaye J, Reisine T, Finkbeiner S. Huntington’s disease iPSC models-using human patient cells to understand the pathology caused by expanded CAG repeats. Fac Rev. 2022;11:16. Epub 20220628. doi: 10.12703/r/11-16 ; PubMed Central PMCID: PMC9264339. 35865413 PMC9264339

[11] Barak M, Fedorova V, Pospisilova V, Raska J, Vochyanova S, Sedmik J, et al. Human iPSC-Derived Neural Models for Studying Alzheimer’s Disease: from Neural Stem Cells to Cerebral Organoids. Stem Cell Rev Rep. 2022;18(2):792–820. Epub 20220202. doi: 10.1007/s12015-021-10254-3 ; PubMed Central PMCID: PMC8930932. 35107767 PMC8930932

[12] Hu X, Mao C, Fan L, Luo H, Hu Z, Zhang S, et al. Modeling Parkinson’s Disease Using Induced Pluripotent Stem Cells. Stem Cells Int. 2020;2020:1061470. Epub 20200312. doi: 10.1155/2020/1061470 ; PubMed Central PMCID: PMC7091557. 32256606 PMC7091557

[13] Gradisnik L, Velnar T. Astrocytes in the central nervous system and their functions in health and disease: A review. World J Clin Cases. 2023;11(15):3385–94. doi: 10.12998/wjcc.v11.i15.3385 ; PubMed Central PMCID: PMC10294192. 37383914 PMC10294192

[14] Robinson C, Apgar C, Shapiro LA. Astrocyte Hypertrophy Contributes to Aberrant Neurogenesis after Traumatic Brain Injury. Neural Plast. 2016;2016:1347987. Epub 20160504. doi: 10.1155/2016/1347987 ; PubMed Central PMCID: PMC4870378. 27274873 PMC4870378

[15] Wilhelmsson U, Li L, Pekna M, Berthold CH, Blom S, Eliasson C, et al. Absence of glial fibrillary acidic protein and vimentin prevents hypertrophy of astrocytic processes and improves post-traumatic regeneration. J Neurosci. 2004;24(21):5016–21. doi: 10.1523/JNEUROSCI.0820-04.2004 ; PubMed Central PMCID: PMC6729371. 15163694 PMC6729371

[16] Savya SP, Li F, Lam S, Wellman SM, Stieger KC, Chen K, et al. In vivo spatiotemporal dynamics of astrocyte reactivity following neural electrode implantation. Biomaterials. 2022;289:121784. Epub 20220902. doi: 10.1016/j.biomaterials.2022.121784 ; PubMed Central PMCID: PMC10231871. 36103781 PMC10231871

[17] Wang J, Sareddy GR, Lu Y, Pratap UP, Tang F, Greene KM, et al. Astrocyte-Derived Estrogen Regulates Reactive Astrogliosis and is Neuroprotective following Ischemic Brain Injury. J Neurosci. 2020;40(50):9751–71. Epub 20201106. doi: 10.1523/JNEUROSCI.0888-20.2020 ; PubMed Central PMCID: PMC7726540. 33158962 PMC7726540

[18] Wang H, Song G, Chuang H, Chiu C, Abdelmaksoud A, Ye Y, et al. Portrait of glial scar in neurological diseases. Int J Immunopathol Pharmacol. 2018;31:2058738418801406. doi: 10.1177/2058738418801406 ; PubMed Central PMCID: PMC6187421. 30309271 PMC6187421

[19] Lagos-Cabre R, Burgos-Bravo F, Avalos AM, Leyton L. Connexins in Astrocyte Migration. Front Pharmacol. 2019;10:1546. Epub 20200115. doi: 10.3389/fphar.2019.01546 ; PubMed Central PMCID: PMC6974553. 32009957 PMC6974553

[20] Tcw J, Wang M, Pimenova AA, Bowles KR, Hartley BJ, Lacin E, et al. An Efficient Platform for Astrocyte Differentiation from Human Induced Pluripotent Stem Cells. Stem Cell Reports. 2017;9(2):600–14. Epub 20170727. doi: 10.1016/j.stemcr.2017.06.018 ; PubMed Central PMCID: PMC5550034. 28757165 PMC5550034

[21] Acharya A, Ambikan AT, Thurman M, Malik MR, Dyavar SR, Vegvari A, et al. Proteomic landscape of astrocytes and pericytes infected with HIV/SARS-CoV-2 mono/co-infection, impacting on neurological complications. Res Sq. 2023. Epub 20230612. doi: 10.21203/rs.3.rs-3031591/v1 ; PubMed Central PMCID: PMC10312942. 37398206 PMC10312942

[22] Magistri M, Khoury N, Mazza EM, Velmeshev D, Lee JK, Bicciato S, et al. A comparative transcriptomic analysis of astrocytes differentiation from human neural progenitor cells. Eur J Neurosci. 2016;44(10):2858–70. Epub 20160925. doi: 10.1111/ejn.13382 ; PubMed Central PMCID: PMC5118072. 27564458 PMC5118072

[23] Perriot S, Mathias A, Perriard G, Canales M, Jonkmans N, Merienne N, et al. Human Induced Pluripotent Stem Cell-Derived Astrocytes Are Differentially Activated by Multiple Sclerosis-Associated Cytokines. Stem Cell Reports. 2018;11(5):1199–210. Epub 20181025. doi: 10.1016/j.stemcr.2018.09.015 ; PubMed Central PMCID: PMC6234919. 30409508 PMC6234919

[24] Chandrasekaran A, Avci HX, Ochalek A, Rosingh LN, Molnar K, Laszlo L, et al. Comparison of 2D and 3D neural induction methods for the generation of neural progenitor cells from human induced pluripotent stem cells. Stem Cell Res. 2017;25:139–51. Epub 20171014. doi: 10.1016/j.scr.2017.10.010 . 29128818

[25] Zhou S, Szczesna K, Ochalek A, Kobolak J, Varga E, Nemes C, et al. Neurosphere Based Differentiation of Human iPSC Improves Astrocyte Differentiation. Stem Cells Int. 2016;2016:4937689. Epub 20151221. doi: 10.1155/2016/4937689 ; PubMed Central PMCID: PMC4699090. 26798357 PMC4699090

[26] Chambers SM, Fasano CA, Papapetrou EP, Tomishima M, Sadelain M, Studer L. Highly efficient neural conversion of human ES and iPS cells by dual inhibition of SMAD signaling. Nature biotechnology. 2009;27(3):275–80. Epub 03/01. doi: 10.1038/nbt.1529 . 19252484 PMC2756723

[27] Shi Y, Kirwan P, Livesey FJ. Directed differentiation of human pluripotent stem cells to cerebral cortex neurons and neural networks. Nature Protocols. 2012;7:1836. doi: 10.1038/nprot.2012.116 22976355

[28] Wei Z, Zhang W, Fang H, Li Y, Wang X. esATAC: an easy-to-use systematic pipeline for ATAC-seq data analysis. Bioinformatics. 2018;34(15):2664–5. doi: 10.1093/bioinformatics/bty141 ; PubMed Central PMCID: PMC6061683. 29522192 PMC6061683

[29] Love MI, Huber W, Anders S. Moderated estimation of fold change and dispersion for RNA-seq data with DESeq2. Genome Biol. 2014;15(12):550. doi: 10.1186/s13059-014-0550-8 ; PubMed Central PMCID: PMC4302049. 25516281 PMC4302049

[30] Galili T. dendextend: an R package for visualizing, adjusting and comparing trees of hierarchical clustering. Bioinformatics. 2015;31(22):3718–20. Epub 20150723. doi: 10.1093/bioinformatics/btv428 ; PubMed Central PMCID: PMC4817050. 26209431 PMC4817050

[31] Gu Z, Hubschmann D. Make Interactive Complex Heatmaps in R. Bioinformatics. 2022;38(5):1460–2. doi: 10.1093/bioinformatics/btab806 ; PubMed Central PMCID: PMC8826183. 34864868 PMC8826183

[32] Sutton GJ, Poppe D, Simmons RK, Walsh K, Nawaz U, Lister R, et al. Comprehensive evaluation of deconvolution methods for human brain gene expression. Nat Commun. 2022;13(1):1358. Epub 20220315. doi: 10.1038/s41467-022-28655-4 ; PubMed Central PMCID: PMC8924248. 35292647 PMC8924248

[33] Hunt GJ, Freytag S, Bahlo M, Gagnon-Bartsch JA. dtangle: accurate and robust cell type deconvolution. Bioinformatics. 2019;35(12):2093–9. doi: 10.1093/bioinformatics/bty926 . 30407492

[34] Zhang Y, Sloan SA, Clarke LE, Caneda C, Plaza CA, Blumenthal PD, et al. Purification and Characterization of Progenitor and Mature Human Astrocytes Reveals Transcriptional and Functional Differences with Mouse. Neuron. 2016;89(1):37–53. Epub 20151210. doi: 10.1016/j.neuron.2015.11.013 ; PubMed Central PMCID: PMC4707064. 26687838 PMC4707064

[35] Moein M, Grzyb K, Goncalves Martins T, Komoto S, Peri F, Crawford AD, et al. CaSiAn: a Calcium Signaling Analyzer tool. Bioinformatics. 2018;34(17):3052–4. doi: 10.1093/bioinformatics/bty281 ; PubMed Central PMCID: PMC6129310. 29668830 PMC6129310

[36] de Leeuw SM, Kirschner AWT, Lindner K, Rust R, Budny V, Wolski WE, et al. APOE2, E3, and E4 differentially modulate cellular homeostasis, cholesterol metabolism, and inflammatory response in isogenic iPSC-derived astrocytes. Stem Cell Reports. 2022;17(1):110–26. Epub 20211216. doi: 10.1016/j.stemcr.2021.11.007 ; PubMed Central PMCID: PMC8758949. 34919811 PMC8758949

[37] Kang P, Lee HK, Glasgow SM, Finley M, Donti T, Gaber ZB, et al. Sox9 and NFIA coordinate a transcriptional regulatory cascade during the initiation of gliogenesis. Neuron. 2012;74(1):79–94. doi: 10.1016/j.neuron.2012.01.024 ; PubMed Central PMCID: PMC3543821. 22500632 PMC3543821

[38] Rajan P, McKay RD. Multiple routes to astrocytic differentiation in the CNS. J Neurosci. 1998;18(10):3620–9. doi: 10.1523/JNEUROSCI.18-10-03620.1998 ; PubMed Central PMCID: PMC6793143. 9570793 PMC6793143

[39] Verkhratsky A, Matteoli M, Parpura V, Mothet JP, Zorec R. Astrocytes as secretory cells of the central nervous system: idiosyncrasies of vesicular secretion. The EMBO Journal. 2016;35(3):239–57-57. doi: 10.15252/embj.201592705 26758544 PMC4741299

[40] Naruse M, Shibasaki K, Yokoyama S, Kurachi M, Ishizaki Y. Dynamic changes of CD44 expression from progenitors to subpopulations of astrocytes and neurons in developing cerebellum. PLoS One. 2013;8(1):e53109. Epub 20130104. doi: 10.1371/journal.pone.0053109 ; PubMed Central PMCID: PMC3537769. 23308146 PMC3537769

[41] Liu Y, Han SS, Wu Y, Tuohy TM, Xue H, Cai J, et al. CD44 expression identifies astrocyte-restricted precursor cells. Dev Biol. 2004;276(1):31–46. doi: 10.1016/j.ydbio.2004.08.018 . 15531362

[42] Gabel S, Koncina E, Dorban G, Heurtaux T, Birck C, Glaab E, et al. Inflammation Promotes a Conversion of Astrocytes into Neural Progenitor Cells via NF-kappaB Activation. Mol Neurobiol. 2016;53(8):5041–55. Epub 20150917. doi: 10.1007/s12035-015-9428-3 ; PubMed Central PMCID: PMC5012156. 26381429 PMC5012156

[43] Lattke M, Goldstone R, Ellis JK, Boeing S, Jurado-Arjona J, Marichal N, et al. Extensive transcriptional and chromatin changes underlie astrocyte maturation in vivo and in culture. Nat Commun. 2021;12(1):4335. Epub 20210715. doi: 10.1038/s41467-021-24624-5 ; PubMed Central PMCID: PMC8282848. 34267208 PMC8282848

[44] Clavreul S, Dumas L, Loulier K. Astrocyte development in the cerebral cortex: Complexity of their origin, genesis, and maturation. Front Neurosci. 2022;16:916055. Epub 20220913. doi: 10.3389/fnins.2022.916055 ; PubMed Central PMCID: PMC9513187. 36177355 PMC9513187

[45] Villarreal A, Vogel T. Different Flavors of Astrocytes: Revising the Origins of Astrocyte Diversity and Epigenetic Signatures to Understand Heterogeneity after Injury. Int J Mol Sci. 2021;22(13). Epub 20210626. doi: 10.3390/ijms22136867 ; PubMed Central PMCID: PMC8268487. 34206710 PMC8268487

[46] Allnoch L, Leitzen E, Zdora I, Baumgartner W, Hansmann F. Astrocyte depletion alters extracellular matrix composition in the demyelinating phase of Theiler’s murine encephalomyelitis. PLoS One. 2022;17(6):e0270239. Epub 20220617. doi: 10.1371/journal.pone.0270239 ; PubMed Central PMCID: PMC9205503. 35714111 PMC9205503

[47] Choi SS, Lee HJ, Lim I, Satoh J, Kim SU. Human astrocytes: secretome profiles of cytokines and chemokines. PLoS One. 2014;9(4):e92325. Epub 20140401. doi: 10.1371/journal.pone.0092325 ; PubMed Central PMCID: PMC3972155. 24691121 PMC3972155

[48] Muir EM, Adcock KH, Morgenstern DA, Clayton R, von Stillfried N, Rhodes K, et al. Matrix metalloproteases and their inhibitors are produced by overlapping populations of activated astrocytes. Brain Res Mol Brain Res. 2002;100(1–2):103–17. doi: 10.1016/s0169-328x(02)00132-8 . 12008026

[49] Perriot S, Canales M, Mathias A, Du Pasquier R. Differentiation of functional astrocytes from human-induced pluripotent stem cells in chemically defined media. STAR Protoc. 2021;2(4):100902. Epub 20211020. doi: 10.1016/j.xpro.2021.100902 ; PubMed Central PMCID: PMC8551928. 34746863 PMC8551928

[50] Voulgaris D, Nikolakopoulou P, Herland A. Generation of Human iPSC-Derived Astrocytes with a mature star-shaped phenotype for CNS modeling. Stem Cell Rev Rep. 2022;18(7):2494–512. Epub 20220430. doi: 10.1007/s12015-022-10376-2 ; PubMed Central PMCID: PMC9489586. 35488987 PMC9489586

[51] Kerkering J, Muinjonov B, Rosiewicz KS, Diecke S, Biese C, Schiweck J, et al. iPSC-derived reactive astrocytes from patients with multiple sclerosis protect cocultured neurons in inflammatory conditions. J Clin Invest. 2023;133(13). Epub 20230703. doi: 10.1172/JCI164637 ; PubMed Central PMCID: PMC10313373. 37219933 PMC10313373

[52] Sullivan MA, Lane SD, McKenzie ADJ, Ball SR, Sunde M, Neely GG, et al. iPSC-derived PSEN2 (N141I) astrocytes and microglia exhibit a primed inflammatory phenotype. J Neuroinflammation. 2024;21(1):7. Epub 20240104. doi: 10.1186/s12974-023-02951-2 ; PubMed Central PMCID: PMC10765839. 38178159 PMC10765839

[53] Williams EC, Zhong X, Mohamed A, Li R, Liu Y, Dong Q, et al. Mutant astrocytes differentiated from Rett syndrome patients-specific iPSCs have adverse effects on wild-type neurons. Hum Mol Genet. 2014;23(11):2968–80. Epub 20140112. doi: 10.1093/hmg/ddu008 ; PubMed Central PMCID: PMC4014193. 24419315 PMC4014193

[54] Hedegaard A, Monzon-Sandoval J, Newey SE, Whiteley ES, Webber C, Akerman CJ. Pro-maturational Effects of Human iPSC-Derived Cortical Astrocytes upon iPSC-Derived Cortical Neurons. Stem Cell Reports. 2020;15(1):38–51. Epub 20200604. doi: 10.1016/j.stemcr.2020.05.003 ; PubMed Central PMCID: PMC7363746. 32502466 PMC7363746

[55] Santos R, Vadodaria KC, Jaeger BN, Mei A, Lefcochilos-Fogelquist S, Mendes APD, et al. Differentiation of Inflammation-Responsive Astrocytes from Glial Progenitors Generated from Human Induced Pluripotent Stem Cells. Stem Cell Reports. 2017;8(6):1757–69. doi: 10.1016/j.stemcr.2017.05.011 ; PubMed Central PMCID: PMC5470172. 28591655 PMC5470172

[56] Meloni M, Morgado J, Garcia M, Stipursky J, Gomes FCA. Cryopreserved astrocytes maintain biological properties: Support of neuronal survival and differentiation. J Neurosci Methods. 2020;343:108806. Epub 20200620. doi: 10.1016/j.jneumeth.2020.108806 . 32574642

[57] Berryer MH, Tegtmeyer M, Binan L, Valakh V, Nathanson A, Trendafilova D, et al. Robust induction of functional astrocytes using NGN2 expression in human pluripotent stem cells. iScience. 2023;26(7):106995. Epub 20230530. doi: 10.1016/j.isci.2023.106995 ; PubMed Central PMCID: PMC10391684. 37534135 PMC10391684

[58] Mulica P, Venegas C, Landoulsi Z, Badanjak K, Delcambre S, Tziortziou M, et al. Comparison of two protocols for the generation of iPSC-derived human astrocytes. Biol Proced Online. 2023;25(1):26. Epub 20230920. doi: 10.1186/s12575-023-00218-x ; PubMed Central PMCID: PMC10512486. 37730545 PMC10512486

[59] Ahlemeyer B, Kolker S, Zhu Y, Hoffmann GF, Krieglstein J. Cytosine arabinofuranoside-induced activation of astrocytes increases the susceptibility of neurons to glutamate due to the release of soluble factors. Neurochem Int. 2003;42(7):567–81. doi: 10.1016/s0197-0186(02)00164-x . 12590940

[60] Neyrinck K, Van Den Daele J, Vervliet T, De Smedt J, Wierda K, Nijs M, et al. SOX9-induced Generation of Functional Astrocytes Supporting Neuronal Maturation in an All-human System. Stem Cell Rev Rep. 2021;17(5):1855–73. Epub 20210512. doi: 10.1007/s12015-021-10179-x ; PubMed Central PMCID: PMC8553725. 33982246 PMC8553725

[61] Li X, Tao Y, Bradley R, Du Z, Tao Y, Kong L, et al. Fast Generation of Functional Subtype Astrocytes from Human Pluripotent Stem Cells. Stem Cell Reports. 2018;11(4):998–1008. Epub 20180927. doi: 10.1016/j.stemcr.2018.08.019 ; PubMed Central PMCID: PMC6178885. 30269954 PMC6178885

[62] Tchieu J, Calder EL, Guttikonda SR, Gutzwiller EM, Aromolaran KA, Steinbeck JA, et al. NFIA is a gliogenic switch enabling rapid derivation of functional human astrocytes from pluripotent stem cells. Nat Biotechnol. 2019;37(3):267–75. Epub 20190225. doi: 10.1038/s41587-019-0035-0 ; PubMed Central PMCID: PMC6591152. 30804533 PMC6591152

[63] Yanez RJ, Porter AC. A chromosomal position effect on gene targeting in human cells. Nucleic Acids Res. 2002;30(22):4892–901. doi: 10.1093/nar/gkf614 ; PubMed Central PMCID: PMC137162. 12433992 PMC137162

[64] Smith AJ, Tasnim N, Psaras Z, Gyamfi D, Makani K, Suzuki WA, et al. Assessing Human Spatial Navigation in a Virtual Space and its Sensitivity to Exercise. J Vis Exp. 2024;(203). Epub 20240126. doi: 10.3791/65332 . 38345261

[65] Escartin C, Galea E, Lakatos A, O’Callaghan JP, Petzold GC, Serrano-Pozo A, et al. Reactive astrocyte nomenclature, definitions, and future directions. Nat Neurosci. 2021;24(3):312–25. Epub 20210215. doi: 10.1038/s41593-020-00783-4 ; PubMed Central PMCID: PMC8007081. 33589835 PMC8007081

[66] Knott JC, Edwards AJ, Gullan RW, Clarke TM, Pilkington GJ. A human glioma cell line retaining expression of GFAP and gangliosides, recognized by A2B5 and LB1 antibodies, after prolonged passage. Neuropathol Appl Neurobiol. 1990;16(6):489–500. doi: 10.1111/j.1365-2990.1990.tb01288.x . 1982895

[67] Nishiyama A, Suzuki R, Zhu X. NG2 cells (polydendrocytes) in brain physiology and repair. Front Neurosci. 2014;8:133. Epub 20140627. doi: 10.3389/fnins.2014.00133 ; PubMed Central PMCID: PMC4072963. 25018689 PMC4072963

[68] Laywell ED, Rakic P, Kukekov VG, Holland EC, Steindler DA. Identification of a multipotent astrocytic stem cell in the immature and adult mouse brain. Proc Natl Acad Sci U S A. 2000;97(25):13883–8. doi: 10.1073/pnas.250471697 ; PubMed Central PMCID: PMC17670. 11095732 PMC17670

[69] Lattke M, Guillemot F. Understanding astrocyte differentiation: Clinical relevance, technical challenges, and new opportunities in the omics era. WIREs Mech Dis. 2022;14(5):e1557. Epub 20220512. doi: 10.1002/wsbm.1557 ; PubMed Central PMCID: PMC9539907. 35546493 PMC9539907

[70] Evans RJ, Wyllie FS, Wynford-Thomas D, Kipling D, Jones CJ. A P53-dependent, telomere-independent proliferative life span barrier in human astrocytes consistent with the molecular genetics of glioma development. Cancer Res. 2003;63(16):4854–61. . 12941806

[71] Pertusa M, Garcia-Matas S, Rodriguez-Farre E, Sanfeliu C, Cristofol R. Astrocytes aged in vitro show a decreased neuroprotective capacity. J Neurochem. 2007;101(3):794–805. Epub 20070123. doi: 10.1111/j.1471-4159.2006.04369.x . 17250685

[72] Kawano H, Katsurabayashi S, Kakazu Y, Yamashita Y, Kubo N, Kubo M, et al. Long-term culture of astrocytes attenuates the readily releasable pool of synaptic vesicles. PLoS One. 2012;7(10):e48034. Epub 20121026. doi: 10.1371/journal.pone.0048034 ; PubMed Central PMCID: PMC3482238. 23110166 PMC3482238

[73] Semyanov A, Henneberger C, Agarwal A. Making sense of astrocytic calcium signals—from acquisition to interpretation. Nat Rev Neurosci. 2020;21(10):551–64. Epub 20200901. doi: 10.1038/s41583-020-0361-8 . 32873937

[74] Fujii Y, Maekawa S, Morita M. Astrocyte calcium waves propagate proximally by gap junction and distally by extracellular diffusion of ATP released from volume-regulated anion channels. Sci Rep. 2017;7(1):13115. Epub 20171013. doi: 10.1038/s41598-017-13243-0 ; PubMed Central PMCID: PMC5640625. 29030562 PMC5640625

[75] de Ceglia R, Ledonne A, Litvin DG, Lind BL, Carriero G, Latagliata EC, et al. Specialized astrocytes mediate glutamatergic gliotransmission in the CNS. Nature. 2023;622(7981):120–9. Epub 20230906. doi: 10.1038/s41586-023-06502-w ; PubMed Central PMCID: PMC10550825. 37674083 PMC10550825

[76] Goenaga J, Araque A, Kofuji P, Herrera Moro Chao D. Calcium signaling in astrocytes and gliotransmitter release. Front Synaptic Neurosci. 2023;15:1138577. Epub 20230302. doi: 10.3389/fnsyn.2023.1138577 ; PubMed Central PMCID: PMC10017551. 36937570 PMC10017551

[77] Wang TF, Zhou C, Tang AH, Wang SQ, Chai Z. Cellular mechanism for spontaneous calcium oscillations in astrocytes. Acta Pharmacol Sin. 2006;27(7):861–8. doi: 10.1111/j.1745-7254.2006.00397.x . 16787570

[78] Lavrentovich M, Hemkin S. A mathematical model of spontaneous calcium(II) oscillations in astrocytes. J Theor Biol. 2008;251(4):553–60. Epub 20080214. doi: 10.1016/j.jtbi.2007.12.011 . 18275973

[79] Vainchtein ID, Molofsky AV. Astrocytes and Microglia: In Sickness and in Health. Trends Neurosci. 2020;43(3):144–54. Epub 20200207. doi: 10.1016/j.tins.2020.01.003 ; PubMed Central PMCID: PMC7472912. 32044129 PMC7472912

[80] Xiong Y, Chen J, Li Y. Microglia and astrocytes underlie neuroinflammation and synaptic susceptibility in autism spectrum disorder. Front Neurosci. 2023;17:1125428. Epub 20230320. doi: 10.3389/fnins.2023.1125428 ; PubMed Central PMCID: PMC10067592. 37021129 PMC10067592

[81] Hyvarinen T, Hagman S, Ristola M, Sukki L, Veijula K, Kreutzer J, et al. Co-stimulation with IL-1beta and TNF-alpha induces an inflammatory reactive astrocyte phenotype with neurosupportive characteristics in a human pluripotent stem cell model system. Sci Rep. 2019;9(1):16944. Epub 20191115. doi: 10.1038/s41598-019-53414-9 ; PubMed Central PMCID: PMC6858358. 31729450 PMC6858358

[82] Pamies D, Sartori C, Schvartz D, Gonzalez-Ruiz V, Pellerin L, Nunes C, et al. Neuroinflammatory Response to TNFalpha and IL1beta Cytokines Is Accompanied by an Increase in Glycolysis in Human Astrocytes In Vitro. Int J Mol Sci. 2021;22(8). Epub 20210414. doi: 10.3390/ijms22084065 ; PubMed Central PMCID: PMC8071021. 33920048 PMC8071021

[83] Roberts AP, Dec K, McAllister B, Tyrrell V, O’Donnell VB, Harwood A, et al. Upregulated NF-κB pathway proteins may underlie APOE44 associated astrocyte phenotypes in sporadic Alzheimer’s disease. bioRxiv. 2023:2023.04.19.537428. doi: 10.1101/2023.04.19.537428

[84] Oberheim NA, Goldman SA, Nedergaard M. Heterogeneity of astrocytic form and function. Methods Mol Biol. 2012;814:23–45. doi: 10.1007/978-1-61779-452-0_3 ; PubMed Central PMCID: PMC3506190. 22144298 PMC3506190

[85] Mayo F, Gonzalez-Vinceiro L, Hiraldo-Gonzalez L, Calle-Castillejo C, Morales-Alvarez S, Ramirez-Lorca R, et al. Aquaporin-4 Expression Switches from White to Gray Matter Regions during Postnatal Development of the Central Nervous System. Int J Mol Sci. 2023;24(3). Epub 20230203. doi: 10.3390/ijms24033048 ; PubMed Central PMCID: PMC9917791. 36769371 PMC9917791

[86] O’Leary LA, Davoli MA, Belliveau C, Tanti A, Ma JC, Farmer WT, et al. Characterization of Vimentin-Immunoreactive Astrocytes in the Human Brain. Front Neuroanat. 2020;14:31. Epub 20200730. doi: 10.3389/fnana.2020.00031 ; PubMed Central PMCID: PMC7406576. 32848635 PMC7406576

[87] Sullivan MA, Lane S, Volkerling A, Engel M, Werry EL, Kassiou M. Three-dimensional bioprinting of stem cell-derived central nervous system cells enables astrocyte growth, vasculogenesis, and enhances neural differentiation/function. Biotechnol Bioeng. 2023;120(10):3079–91. Epub 20230703. doi: 10.1002/bit.28470 . 37395340 PMC10953436

