# Supplementary material for: Efficient derivation of functional astrocytes from human induced pluripotent stem cells (hiPSCs)
Source: PLoS One. 2024 Dec 4;19(12):e0313514. doi: 10.1371/journal.pone.0313514 (PMC11616838; doi:10.1371/journal.pone.0313514)
Supplement: S1 Table — (PDF) [file pone.0313514.s001.pdf]

S1 Table. Primary antibodies used for immunocytochemistry.

Supplier	Name (dilution)	cat#	Lot#	Host
Abcam	Anti-GFAP (1:1000)	ab4674	GR3234435-1	Chicken, monoclonal
Invitrogen	Glutamine-Synthetase (1:500)	MA5-27749	YE3918926A	mouse, monoclonal
Novus	anti-NFIA (1:250)	NBP181406	44688	rabbit, polyclonal
EMD, Millipore	Anti-ALDHL1 (1:100)	MABN495	3934343	mouse monoclonal
Cell Signaling Technology	Vimentin (1:100)	5741s	1	rabbit IgG
Abcam	S100-beta (1:1000)	AB11178	GR313020-3	mouse IgG
Abcam	Anti-Aqp4 (1:100)	Abcam	GR3214439-1	mouse igG3
