# Supplementary material for: Efficient derivation of functional astrocytes from human induced pluripotent stem cells (hiPSCs)
Source: PLoS One. 2024 Dec 4;19(12):e0313514. doi: 10.1371/journal.pone.0313514 (PMC11616838; doi:10.1371/journal.pone.0313514)
Supplement: S2 Table — (PDF) [file pone.0313514.s002.pdf]

S2 Table. Antibodies used for Western blot.

Antibody	Dilution	Manufacturer	Cat#
Primary antibodies			
AQP4	1:1000	Cell Signalling	59678
VIM	1:1000	Cell Signalling	5741
GFAP	1:1000	Cell Signalling	12389
β-tubulin	1:1000	Cell Signalling	2146
ALDH1L1	1:2000	Abcam	ab177463
Secondary antibody			
Anti-rabbit IgG, HRP	1:1000	Cell Signalling	7074S
