# Supplementary material for: Efficient derivation of functional astrocytes from human induced pluripotent stem cells (hiPSCs)
Source: PLoS One. 2024 Dec 4;19(12):e0313514. doi: 10.1371/journal.pone.0313514 (PMC11616838; doi:10.1371/journal.pone.0313514)
Supplement: S3 Table — (PDF) [file pone.0313514.s003.pdf]

S3 Table. List of controls and cytokines/chemokines analyzed, indicating their location on the Cytokine Array.

	Gene	Alternate Nomenclature	Entrez Gene ID
A1, A2	Reference Spots	RS	N/A
A3, A4	Adiponectin	Acrp30	9370
A5, A6	Apolipoprotein A-I	ApoA1	335
A7, A8	Angiogenin	-	283
A9, A10	Angiopoietin-1	Ang-1, ANGPT1	284
A11, A12	Angiopoietin-2	Ang-2, ANGPT2	285
A13, A14	BAFF	BLyS, TNFSF13B	10673
A15, A16	BDNF	Brain-derived Neurotrophic Factor	627
A17, A18	Complement Component	C5/C5a	727
A19, A20	CD14	-	929
A21, A22	CD30	TNFRSF8	943
A23, A24	Reference Spots	RS	N/A
B3, B4	CD40 ligand	CD40L, TNFSF5, CD154, TRAP	959
B5, B6	Chitinase 3-like 1	CHI3L1, YKL-40	1116
B7, B8	Complement Factor D	Adipsin, CFD	1675
B9, B10	C-Reactive Protein	CRP	1401
B11, B12	Cripto-1	Teratocarcinoma-derived Growth Factor	6997
B13, B14	Cystatin C	CST3, ARMD11	1471
B15, B16	Dkk-1	Dickkopf-1	22943
B17, B18	DPPIV	CD26, DPP4, Dipeptidyl-peptidase IV	1803
B19, B20	EGF	Epidermal Growth Factor	1950
B21, B22	EMMPRIN	CD147, Basigin	682
C3, C4	ENA-78	CXCL5	6374
C5, C6	Endoglin	CD105, ENG	2022
C7, C8	Fas Ligand	TNFSF6, CD178, CD95L	356
C9, C10	FGF basic	FGF-2	2247
C11, C12	FGF-7	KGF	2252
C13, C14	FGF-19	-	9965
C15, C16	Flt-3 Ligand	FLT3LG	2323
C17, C18	G-CSF	CSF3	1440
C19, C20	GDF-15	MIC-1	9518
C21, C22	GM-CSF	CSF2	1437
D1, D2	GROα	CXCL1, MSGA-α	2919
D3, D4	Growth Hormone	GH, Somatotropin	2688
D5, D6	HGF	Scatter Factor, SF	3082
D7, D8	ICAM-1	CD54	3383
D9, D10	IFN-γ	IFNG	3458
D11, D12	IGFBP-2	-	3485
D13, D14	IGFBP-3	-	3486
D15, D16	IL-1α	IL-1F1	3552
D17, D18	IL-1β	IL-1F2	3553
D19, D20	IL-1ra	IL-1F3	3557
D21, D22	IL-2	-	3558
D23, D24	IL-3	-	3562
E1, E2	IL-4	-	3565
E3, E4	IL-5	-	3567
E5, E6	IL-6	-	3569
E7, E8	IL-8	CXCL8	3576

E9, E10	IL-10	-	3586
E11, E12	IL-11	-	3589
E13, E14	IL-12 p70	-	3593
E15, E16	IL-13	-	3596
E17, E18	IL-15	-	3600
E19, E20	IL-16	-	3603
E21, E22	IL-17A	IL-17, CTLA8	3605
E23, E24	IL-18 Bpa	-	10068
F1, F2	IL-19	-	29949
F3, F4	IL-22	IL-TIF	50616
F5, F6	IL-23	IL-23A, SGRF	51561
F7, F8	IL-24	C49A, FISP, MDA-7, MOB-5, ST16	11009
F9, F10	IL-27	-	246778
F11, F12	IL-31	-	386653
F13, F14	IL-32	-	9235
F15, F16	IL-33	C9orf26, DVS27, NF-HEV	90865
F17, F18	IL-34	C16orf77	146433
F19, F20	IP-10	CXCL10	3627
F21, F22	I-TAC	CXCL11, SCYB9B	6373
F23, F24	Kallikrein 3	PSA, KLK3	354
G1, G2	Leptin 3952 O	OB	3952
G3, G4	LIF	-	3976
G5, G6	Lipocalin-2	NGAL, LCN2, Siderocalin	3934
G7, G8	MCP-1	CCL2, MCAF	6347
G9, G10	MCP-3	CCL7, MARC	6354
G11, G12	M-CSF	CSF1	1435
G13, G14	MIF	-	4282
G15, G16	MIG	CXCL9	4283
G17, G18	MIP-1α/MIP-1β	CCL3/CCL4	6348/6351
G19, G20	MIP-3α	CCL20, Exodus-1, LARC	6364
G21, G22	MIP-3β	CCL19, ELC	6363
G23, G24	MMP-9	CLG4B, Gelatinase B	4318
H1, H2	Myeloperoxidase	MPO, Lactoperoxidase	4353
H3, H4	Osteopontin	OPN	6696
H5, H6	PDGF-AA	-	5154
H7, H8	PDGF-AB/BB	-	5154/5155
H9, H10	Pentraxin 3	PTX3, TSG-14	5806
H11, H12	PF4	CXCL4	5196
H13, H14	RAGE	-	177
H15, H16	RANTES	CCL5	6352
H17, H18	RBP-4	-	5950
H19, H20	Relaxin-2	RLN2, RLX	6019
H21, H22	Resistin	ADSF, FIZZ3, RETN	56729
H23, H24	SDF-1α	CXCL12, PBSF	6387
I1, I2	Serpin E1	PAI-I, PAI-1, Nexin	5054
I3, I4	SHBG	ABP	6462
I5, I6	ST2	IL-1 R4, IL1RL1, ST2L	9173
I7, I8	TARC	CCL17	6361
I9, I10	TFF3	ITF, TFI	7033
I11, I12	TfR	CD71, TFR1, TFRC, TRFR	7037
I13, I14	TGF-α	TGFA	7039
I15, I16	Thrombospondin-1	THBS1, TSP-1	7057
I17, I18	TNF-α	TNFSF1A	7124
I19, I20	uPAR	PLAUR	5329
I21, I22	VEGF	BEGFA	7422

J1, J2	Reference Spots	RS	N/A
J5, J6	Vitamin D BP	VDB, DBP, VDBP	2638
J7, J8	CD31	PECAM-1	5175
J9, J10	TIM-3	HAVCR2	84868
J11, J12	VCAM-1	CD106	7412
J23, J24	Negative Controls	Control (-)	N/A
