# Supplementary figures and images for: Efficient derivation of functional astrocytes from human induced pluripotent stem cells (hiPSCs)
Source: PLoS One. 2024 Dec 4;19(12):e0313514. doi: 10.1371/journal.pone.0313514 (PMC11616838; doi:10.1371/journal.pone.0313514)

**S1 Fig. Original uncropped Western blot images.**

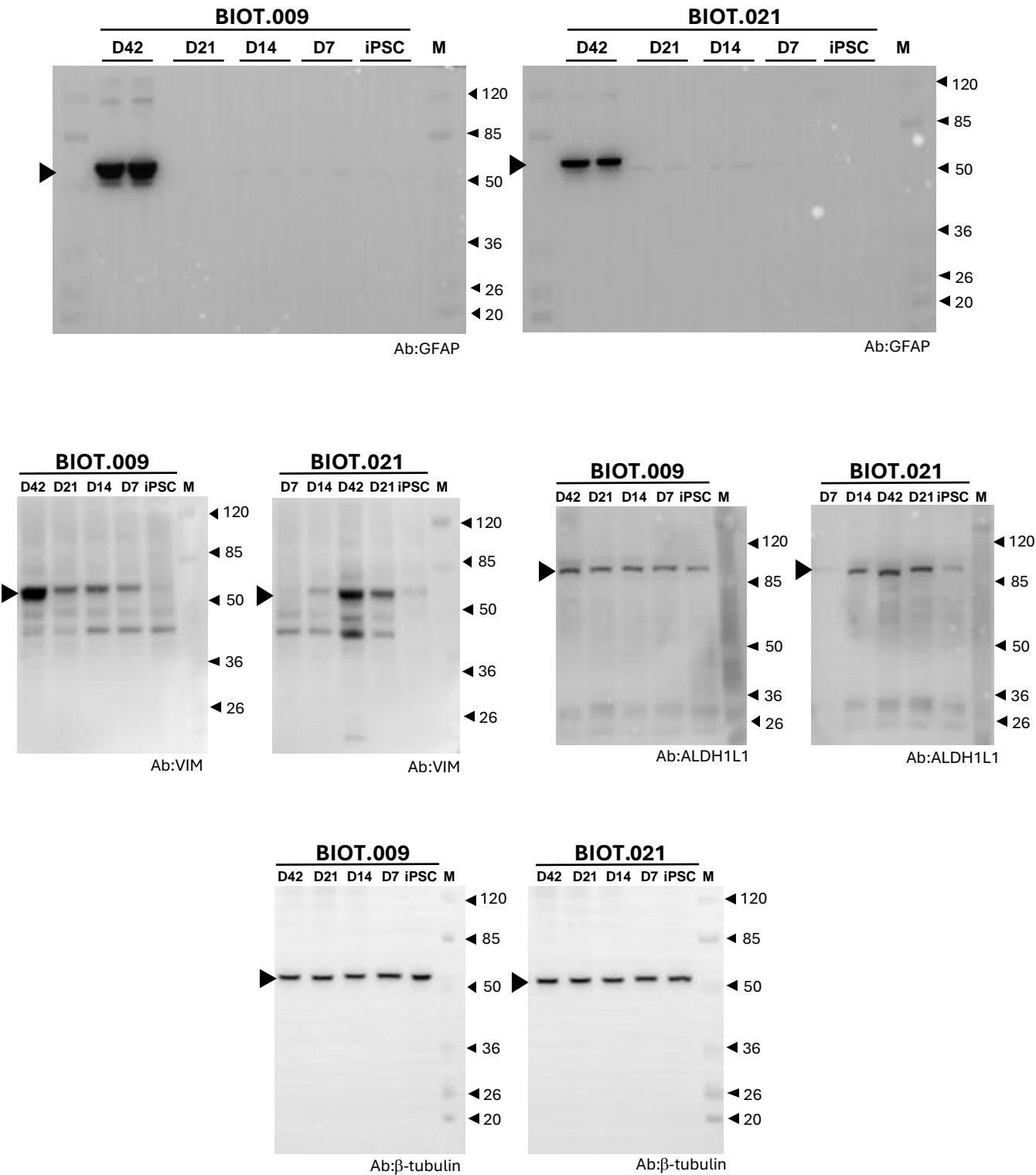

Supplement: S1 Fig — (PDF) [file pone.0313514.s004.pdf]

**S3 Fig. Expression of mature astrocyte markers in Day 21 astroglial progenitor cells.**

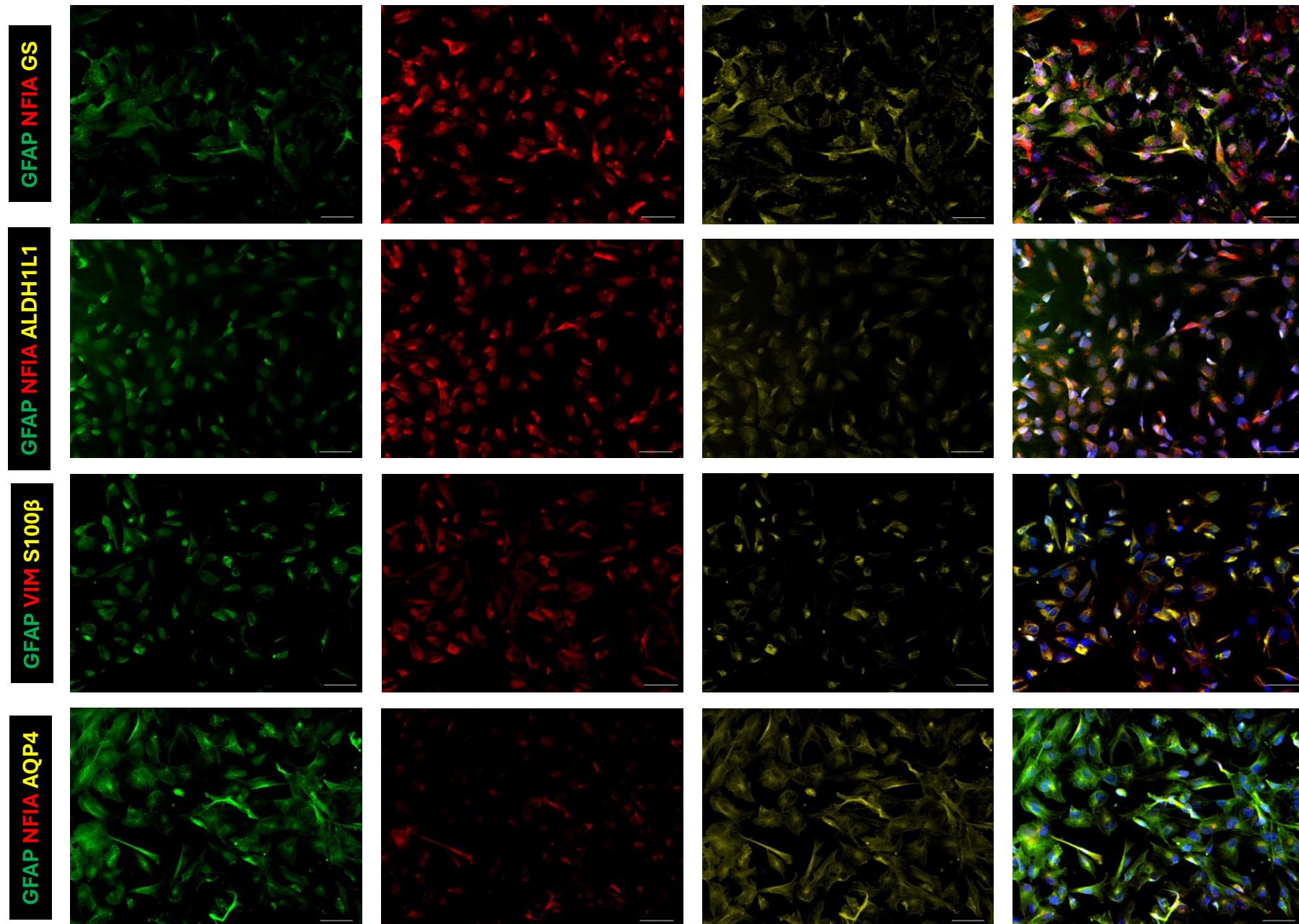

Scale bar: 50  $\mu$ m

Supplement: S3 Fig — (PDF) [file pone.0313514.s006.pdf]

**S4 Fig. Expression of astroglial progenitor markers in Day 42 astrocyte cultures.**

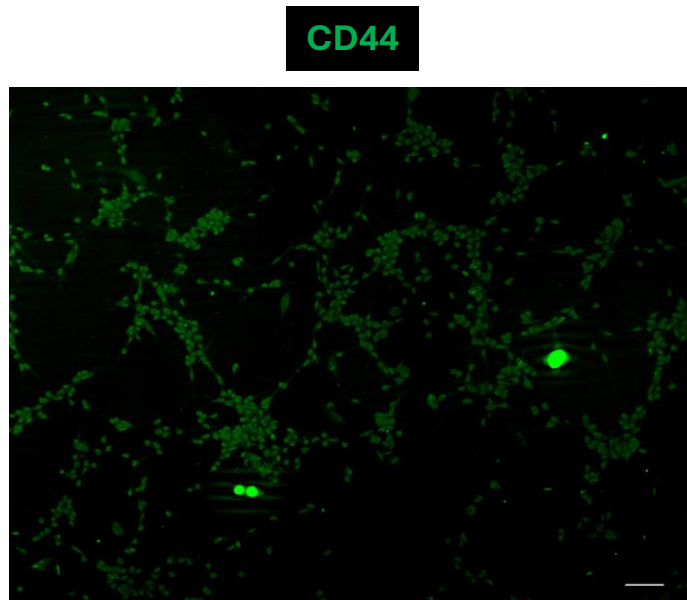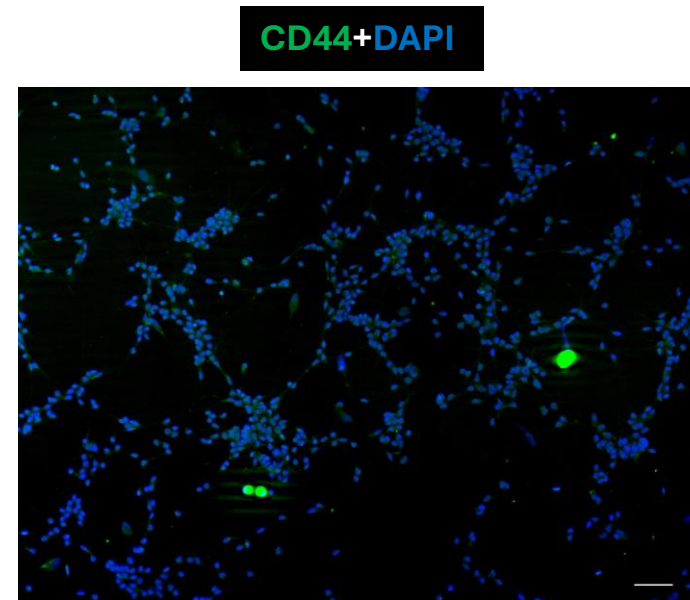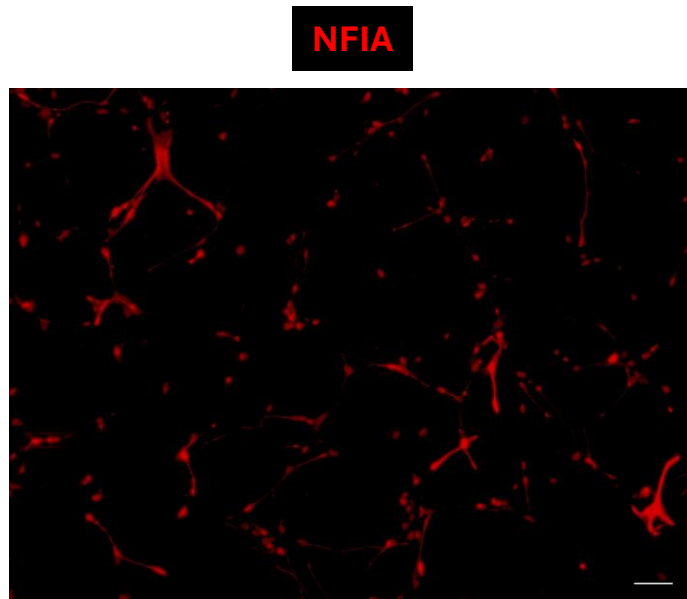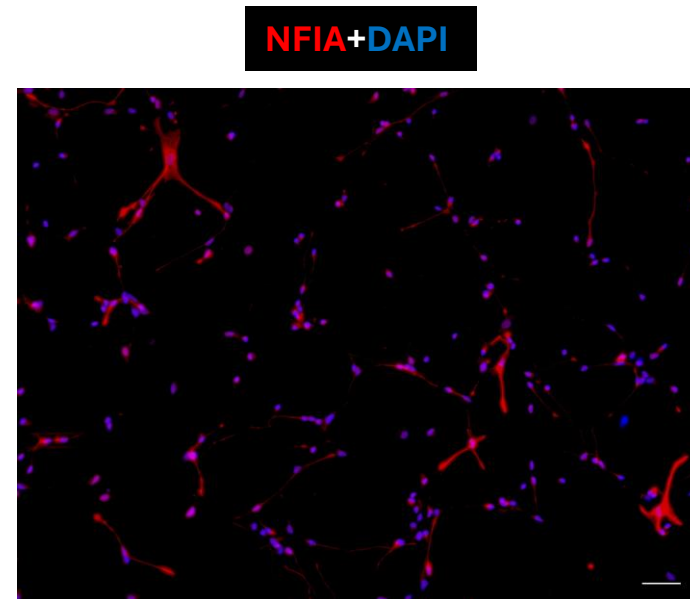

Scale bar: 50  $\mu$ m

Supplement: S4 Fig — (PDF) [file pone.0313514.s007.pdf]
