# Supplementary material for: Efficient derivation of functional astrocytes from human induced pluripotent stem cells (hiPSCs)
Source: PLoS One. 2024 Dec 4;19(12):e0313514. doi: 10.1371/journal.pone.0313514 (PMC11616838; doi:10.1371/journal.pone.0313514)
Supplement: S2 Fig — (PDF) [file pone.0313514.s005.pdf]

S2 Fig. Negative control immunostainings with the secondary antibodies used in the study.

Goat anti-chicken AF488
Donkey anti-rabbit AF594
Donkey anti-mouse AF647

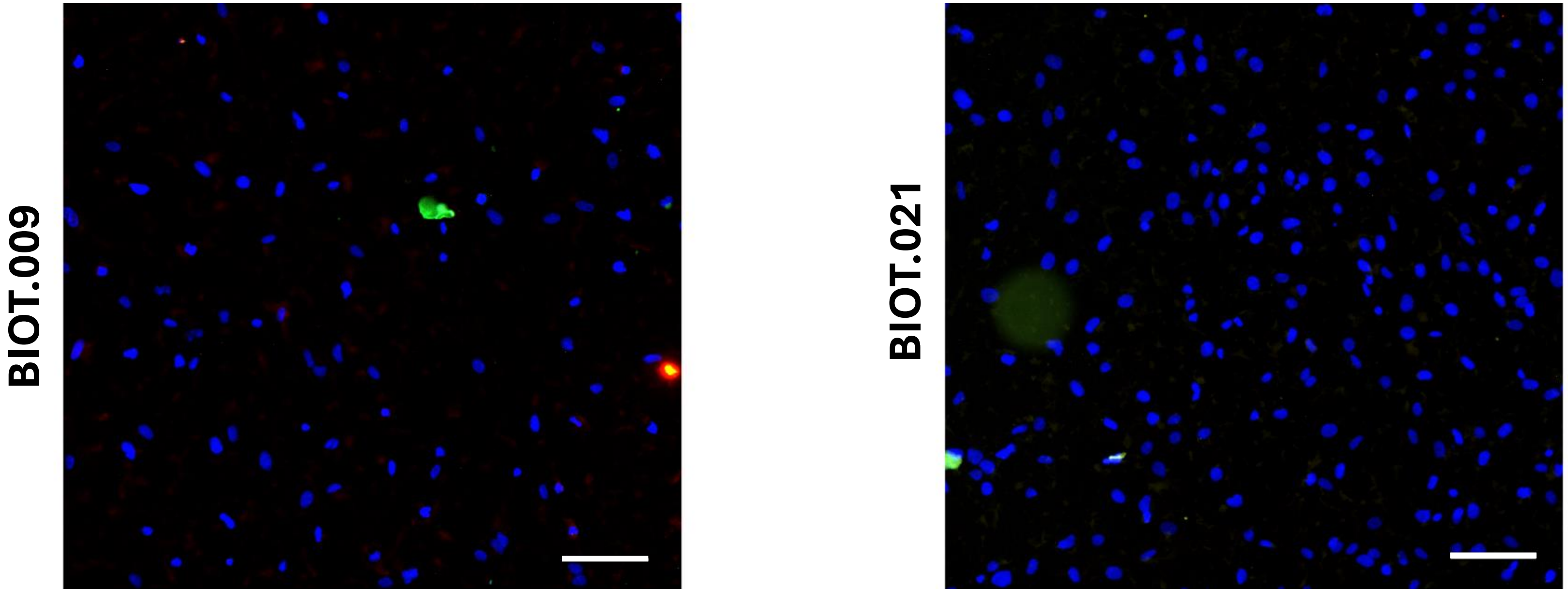

Scale bar: 50 µm
